# Glycolipid‐peptide conjugate vaccines elicit CD8 ^+^ T‐cell responses and prevent breast cancer metastasis

**DOI:** 10.1002/cti2.1401

**Published:** 2022-07-03

**Authors:** Olivia K Burn, Kathryn Farrand, Tara Pritchard, Sarah Draper, Ching‐wen Tang, Anna H Mooney, Alfonso J Schmidt, Sung H Yang, Geoffrey M Williams, Margaret A Brimble, Matheswaran Kandasamy, Andrew J Marshall, Kate Clarke, Gavin F Painter, Ian F Hermans, Robert Weinkove

**Affiliations:** ^1^ Malaghan Institute of Medical Research Wellington New Zealand; ^2^ Department of Pathology & Molecular Medicine University of Otago Wellington Wellington New Zealand; ^3^ Ferrier Research Institute Victoria University of Wellington Wellington New Zealand; ^4^ School of Chemical Sciences University of Auckland Auckland New Zealand; ^5^ School of Biological Sciences University of Auckland Auckland New Zealand; ^6^ Maurice Wilkins Centre Auckland New Zealand; ^7^ Medical Research Council Human Immunology Unit, Weatherall Institute of Molecular Medicine University of Oxford Oxford UK; ^8^ Wellington Blood & Cancer Centre Capital & Coast District Health Board Wellington New Zealand

**Keywords:** breast cancer, cancer vaccine, glycolipid, metastasis, NKT cells, oncogene protein HER‐2, peptide, T cell, triple‐negative breast cancer

## Abstract

**Objectives:**

Metastasis is the principal cause of breast cancer mortality. Vaccines targeting breast cancer antigens have yet to demonstrate clinical efficacy, and there remains an unmet need for safe and effective treatment to reduce the risk of metastasis, particularly for people with triple‐negative breast cancer (TNBC). Certain glycolipids can act as vaccine adjuvants by specifically stimulating natural killer T (NKT) cells to provide a universal form of T‐cell help.

**Methods:**

We designed and made a series of conjugate vaccines comprising a prodrug of the NKT cell‐activating glycolipid α‐galactosylceramide covalently linked to tumor‐expressed peptides, and assessed these using E0771‐ and 4T1‐based breast cancer models *in vivo*. We employed peptides from the model antigen ovalbumin and from clinically relevant breast cancer antigens HER2 and NY‐ESO‐1.

**Results:**

Glycolipid‐peptide conjugate vaccines that activate NKT cells led to antigen‐presenting cell activation, induced inflammatory cytokines, and, compared with peptide alone or admixed peptide and α‐galactosylceramide, specifically enhanced CD8^+^ T‐cell responses against tumor‐associated peptides. Primary tumor growth was delayed by vaccination in all tumor models. Using 4T1‐based cell lines expressing HER2 or NY‐ESO‐1, a single administration of the relevant conjugate vaccine prevented tumor colonisation of the lung following intravenous inoculation of tumor cells or spontaneous metastasis from breast, respectively.

**Conclusion:**

Glycolipid‐peptide conjugate vaccines that activate NKT cells prevent lung metastasis in breast cancer models and warrant investigation as adjuvant therapies for high‐risk breast cancer.

## Graphical abstract

In this study, we found that glycolipid‐peptide conjugate vaccines elicited greater peptide‐specific CD8^+^ T cell responses than peptide ± GM‐CSF and inhibited breast cancer metastasis to lung.

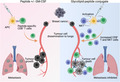

## Introduction

Breast cancer is the most frequent malignancy worldwide, accounting for nearly 12% of global cancer diagnoses in 2020.^1^ Early diagnosis and resection are the mainstay of breast cancer treatment, but metastasis to distal sites such as lung, bone, liver or brain can occur years after initial treatment and is the principal cause of breast cancer‐related mortality.^2^

Adjuvant therapies, including chemotherapy, radiotherapy, hormonal agents and passive (antibody‐based) immunotherapy directed against human epidermal growth factor receptor 2 (HER2), reduce the rate of late metastasis and prolong survival in women with high‐risk breast cancer. However, relapse still occurs in some individuals, and adjuvant options for women with triple‐negative breast cancer (TNBC), which lacks expression of oestrogen receptors, progesterone receptors and HER2, are particularly limited. Moreover, antibody‐based treatments directed against surface HER2, an antigen overexpressed in about 15–30% of breast cancers,^3^, ^4^, ^5^, ^6^ are limited by resistance mechanisms including the expression of truncated HER2 variants and shedding of the HER2 ectodomain.^7^ There remains an unmet need for effective and safe adjuvant therapies that reduce the risk of late metastasis.^8^

Vaccines directed against tumor‐associated antigens have the potential to elicit T cell‐mediated antitumor activity, even if the intact antigen is not expressed on the tumor cell surface. Fragments of target antigens can be processed intracellularly and presented to cytotoxic CD8^+^ T cells. Peptide vaccines directed against HER2, including HER2_369–377_ (E7; nelipepimut‐S (NP‐S;) NeuVax), HER2_654–662_ (GP2) and li‐Key/HER2_776–790_ (AE37; a hybrid of the li‐Key and HER2_776–790_ peptides), have all elicited anti‐HER2 T‐cell responses in clinical trials, but none have yet demonstrated unequivocal clinical efficacy.^9^, ^10^, ^11^, ^12^

One reason for the lack of clinical responses to HER2 peptide vaccines may be the selection of vaccine adjuvants.^13^ Vaccine adjuvants capable of enhancing cytotoxic T‐cell responses include ligands for pattern recognition receptors such as Toll‐like receptors (TLRs) and maturation‐inducing cytokines such as granulocyte macrophage‐colony‐stimulating factor (GM‐CSF). Peptide sequences that bind to MHC class II to recruit helper CD4^+^ T cells can also enhance cytotoxic responses. Helper activity can also be provided by compounds that activate invariant natural killer T (NKT) cells, a class of T‐cell that specifically recognises glycolipids rather than peptides.^14^, ^15^, ^16^, ^17^, ^18^ By co‐administering an antigenic peptide with the archetypal NKT cell‐activating glycolipid, α‐galactosylceramide (α‐GalCer), both CD8^+^ and CD4^+^ T‐cell responses against the peptide are enhanced *in vivo*.^17^, ^18^ We have shown that covalently linking a prodrug form of α‐GalCer to a peptide antigen further enhances cytotoxic T‐cell responses, potentially by assuring delivery of the target peptide and vaccine adjuvant to the same antigen‐presenting cell (APC).^19^, ^20^ These glycolipid‐peptide conjugate vaccines can elicit protective T‐cell resident memory,^21^ and enhance human T‐cell responses against viral antigens.^22^

We hypothesised that NKT cell‐activating glycolipid‐peptide conjugates could enhance responses to peptide vaccination in models of breast cancer. In this study, we first demonstrate the efficacy of glycolipid‐peptide conjugate vaccines in an ovalbumin‐expressing E0771 breast cancer model in C57BL/6J mice. Next, we assess this strategy in BALB/cJ mice, which have a different phenotypic profile of NKT cells to that of C57BL/6J mice using two 4T1‐based breast cancer models.^23^, ^24^, ^25^ We report that a single dose of a glycolipid‐peptide conjugate vaccine directed against the clinically relevant antigen HER2 delays tumor growth and prevents tumor colonisation of the lung in an experimental metastasis model. Finally, we demonstrate efficacy of a third glycolipid‐peptide conjugate vaccine in a spontaneous metastasis model, targeting a tumor‐associated antigen frequently overexpressed in triple‐negative breast cancer (TNBC), New York oesophageal squamous cell carcinoma (NY‐ESO‐1).^26^, ^27^, ^28^ Our results suggest that glycolipid‐peptide conjugate vaccines warrant further investigation for the adjuvant treatment of high‐risk breast cancer.

## Results

### Vaccines utilising the cellular adjuvant properties of NKT cells induce antitumor activity in E0771 model of breast cancer

**Figure 1:**
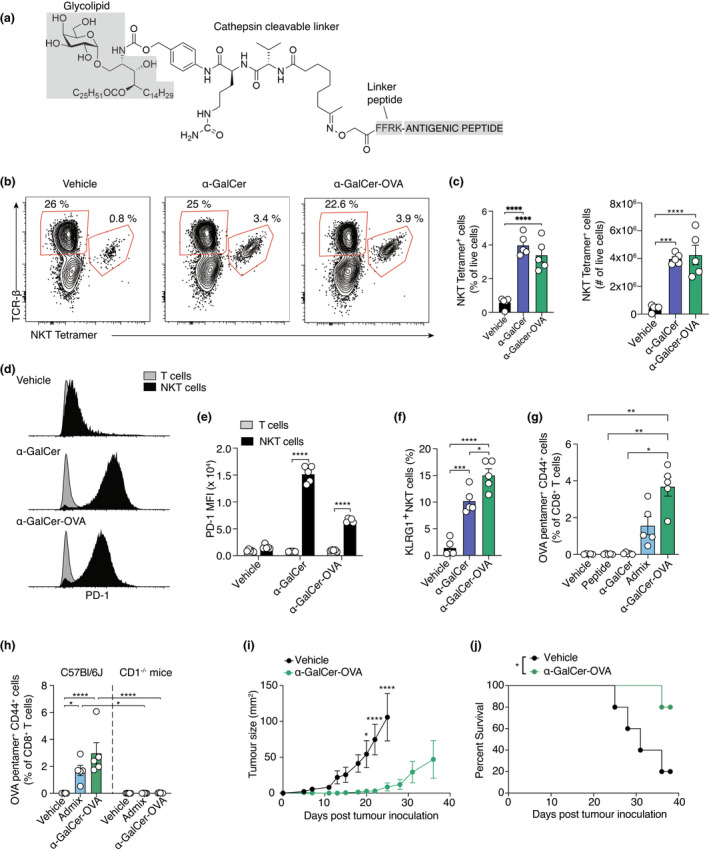
Glycolipid‐peptide vaccine targeting OVA activates NKT cells, elicits a CD8^+^ T‐cell response and protects against E0771^−^OVA breast cancer growth *in vivo*. **(a)** Generic chemical structure of α‐GalCer‐peptide conjugate vaccines. For this experiment, the antigenic peptide conjugated to α‐GalCer was the synthetic OVA long peptide. **(b)** Typical flow cytometry plots depicting splenic PBS‐57‐loaded CD1d tetramer^+^ (NKT tetramer^+^) T cells at day 7 post‐administration of 3 nmol α‐GalCer‐OVA, α‐GalCer or vehicle (PBS) in C57BL/6J mice. In text is the frequency of NKT tetramer^+^ or NKT tetramer^−^ T cells from total live cells. **(c)** The frequency and number of NKT tetramer^+^ cells of total live splenic cells, shown as mean ± SEM. NKT cells, were defined as PBS‐57‐loaded CD1d tetramer^+^ TCR‐β^+^ CD64^−^ CD19^−^ cells. The results of one of three independent experiments are shown. **(d)** Expression of PD‐1 on NKT tetramer^+^ and NKT tetramer^−^ T cells. **(e)** Mean fluorescent intensity (MFI) of PD‐1 on the various groups. Symbols represent individual mice. Mean ± SEM for each group are shown. **(f)** Expression of KLRG1 on NKT cells. **(g)** Frequency of OVA‐specific CD8^+^ T cells in the peripheral blood 7 days after immunisation with 0.5 nmol of the indicated compounds, assessed by flow cytometry using a H‐2K^b^/OVA_257–264_ pentamer to gate peptide‐specific cells. **(h)** Frequency of OVA‐specific CD8^+^ T cells in the peripheral blood of C57Bl/6J or CD1^−/−^ mice 7 days after immunisation with 0.5 nmol of the indicated compounds, assessed by flow cytometry using a H‐2K^b^/OVA_257–264_ pentamer to gate peptide‐specific cells. **(i)** Tumor growth in mice injected S.C. with 5 × 10^5^ E0771‐OVA cells 7 days after I.V. administration of 2 nmol α‐GalCer‐OVA. Mean tumor volume ± SEM; 5 animals per group. **(j)** Kaplan–Meier survival curves showing survival to prespecified endpoint. For all experiments, *P < 0.05, **P < 0.01, ***P < 0.001, ****P < 0.0001; **(c, f, g, h)** one‐way ANOVA with Tukey's multiple comparison test; **(e)** two‐way ANOVA with Tukey's multiple comparison test; **(h)** two‐way ANOVA with Sidak's multiple comparison test; **(i)** Gehan‐Breslow‐Wilcoxon test.

To assess the cellular adjuvant activity of NKT cells in driving T‐cell responses against tumor‐associated antigens in a breast cancer model, we first synthesised an NKT cell‐activating vaccine designed to raise responses to ovalbumin (OVA), expressed as a model neoantigen. The vaccine comprised an OVA peptide containing the defined CD8^+^ T‐cell epitope OVA_257‐263_ conjugated *via* an enzymatically cleavable linker to an inactive prodrug form of α‐GalCer.^19^ The final vaccine, designated α‐GalCer‐OVA (Figure 1a), is designed to be cleaved by cathepsins after cellular uptake *in vivo*, with the linker immolating, releasing the prodrug and peptide within the same antigen‐presenting cells (APC).^20^ Without the attached linker, the prodrug readily reverts to α‐GalCer, which can be presented *via* CD1d to NKT cells.^19^

To show the vaccine functioned as expected, we first assessed whether there was an NKT cell response to α‐GalCer‐OVA in C57BL/6J mice after I.V. injection. On day 7 after vaccination, a greater than threefold increase in NKT cells was evident in α‐GalCer‐OVA‐treated mice than that in vehicle (PBS)‐treated mice, as assessed by flow cytometry using PBS‐57‐loaded CD1d tetramers (Figure 1b and c). Vaccine‐induced NKT cell expansion was similar to that observed in mice injected with α‐GalCer alone, but expression of the negative T‐cell regulator programmed death‐1 (PD‐1) was lower (Figure 1d and e), and expression of Killer cell lectin‐like receptor subfamily G member 1 (KLRG1), a marker associated with long‐term effector function,^29^, ^30^, ^31^ was higher (Figure 1f).

We then assessed peptide‐specific CD8^+^ T‐cell responses to the vaccine. Administration of peptide alone failed to elicit a detectable peptide‐specific T‐cell response, as determined by the frequency of circulating OVA‐pentamer^+^ CD8^+^ T cells (Figure 1g). While injection of an admix of α‐GalCer and OVA_257–264_ peptide was sufficient to induce a T‐cell response, significantly greater responses were induced with the conjugate vaccine at equimolar concentrations (Figure 1g). To determine whether this activity was dependent on NKT cells, analysis was also conducted in CD1d‐deficient (*CD1d* ^−/−^) animals, which are devoid of all CD1d‐restricted T cells, including NKT cells.^32^ The OVA‐specific CD8^+^ T‐cell response was lost in mice lacking CD1d expression, suggesting NKT cells are involved in the process of peptide‐specific CD8^+^ T‐cell priming (Figure 1h).

Next, we assessed the effect of vaccination on subcutaneous growth of OVA‐expressing E0771 (E0771‐OVA), a murine cell line employed as a model of the luminal B subtype of breast cancer.^33^ The conjugate vaccine significantly delayed growth of E0771‐OVA (1/5 alive vs 4/5 alive at endpoint; *P* = 0.0398) when breast tumor cells were injected seven days after vaccination (Figure 1i and j).

### Early immune response to glycolipid vaccines in BALB/c mice

To extend our findings to a second breast cancer model, we utilised the 4T1.2‐HER2 cell line, an epithelial murine breast cancer model on a BALB/cJ background that has been engineered to express human HER2.^34^ BALB/cJ mice have a twofold lower frequency of hepatic NKT cells than C57BL/6J mice (the hosts used for E0771‐OVA) and a greater frequency of type 2 NKT (NKT2) cells than in type 1 NKT (NKT1) cells, resulting in a cytokine profile that features higher levels of IL‐4.^23^, ^24^ In this respect, the NKT cell population in BALB/cJ mice resembles that in humans, which exhibit low NKT frequencies with a predominance of NKT2 cells.^35^, ^36^

**Figure 2:**
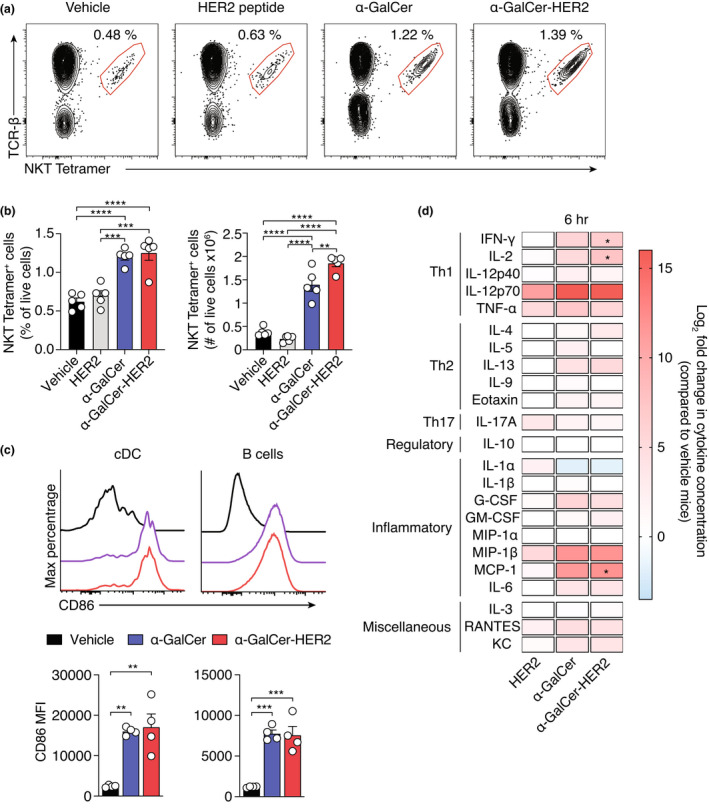
α‐GalCer‐HER2 vaccine activates NKT cells and APCs and elicits inflammatory cytokines in BALB/cJ mice. **(a)** Mice were administered 20 nmol of α‐GalCer‐HER2 or vehicle (PBS) and the activation status of NKT was assessed 7 days later in the spleen. Typical flow cytometry plots depicting splenic PBS‐57‐loaded CD1d tetramer^+^ TCR‐β^+^ T cells following the indicated treatments. The NKT cell frequency from total live cells is shown. **(b)** Frequency and number of splenic NKT cells of total live cells for each treatment group. **(c)** Expression of CD86 on splenic cDC (B220^−^ CD11c^+^ MHC‐II^+^), and B cells (B220^+^ CD11c^−^ MHC‐II^+^). **(d)** Serum cytokines at 6 h after administration, as determined by Bio‐plex multiplex immunoassay. Samples were normalised to PBS treated animals. *denotes statistical difference between the α‐GalCer‐HER2 and the HER2 only group. **(b, c)** One‐way ANOVA with Tukey's multiple comparison test *P < 0.05, ***P < 0.001; **(** **d** **)** One‐way ANOVA with Bonferroni correction *P < 0.002.

To assess antitumor activity of the glycolipid‐peptide conjugate vaccine design in this model, we manufactured and evaluated responses to a construct incorporating the peptide sequence HER2_63‐71_, which is presented on H‐2K^d^ in BALB/cJ mice (α‐GalCer‐HER2). Administration of this vaccine induced significant increases in NKT cells, measured in spleen after 7 days, reaching levels that were slightly higher than when α‐GalCer was administered (1.9 × 10^6^ vs 1.4 × 10^6^ mean NKT cell number; *P* = 0.0075) (Figure 2a and b). As the adjuvant effect of α‐GalCer requires conditioning of dendritic cells (DCs) by NKT cells,^17^, ^18^ we assessed expression of the activation marker CD86 on splenic APCs by flow cytometry 18 h after administration, using the gating strategy in Supplementary figure 2. Significantly increased expression of CD86 was observed on conventional DCs and B cells (Figure 2c), reaching levels similar to those elicited by free α‐GalCer at equimolar concentrations (Figure 2c). As a further readout of NKT cell activity, the cytokine profile in plasma was also assessed (Figure 2d). Compared with PBS control, injection of HER2_63‐71_ peptide alone resulted in increased production of IL‐12p70, TNF‐α, and macrophage inflammatory protein (MIP)‐1β at 6 h (Figure 2d). However, the α‐GalCer‐HER2 vaccine led to a significantly enhanced cytokine profile, featuring increases in Th1‐like cytokines including IFN‐γ, IL‐2 and IL‐12p70, as well as Th2‐like cytokines IL‐13 and eotaxin (Figure 2d). This profile of cytokine expression was similar to that of free α‐GalCer. Both α‐GalCer and α‐GalCer‐HER2 also increased inflammatory chemokines, with large increases in MIP‐1β and MCP‐1 compared to controls. Together, these results indicate that the glycolipid‐peptide conjugate vaccine has strong immunostimulatory activity for NKT cells in BALB/cJ mice and suggest it is effectively processed to release the NKT cell agonist *in vivo*, leading to downstream licensing of DCs.

### Vaccines have antitumor activity in 4T1 model of breast cancer

**Figure 3:**
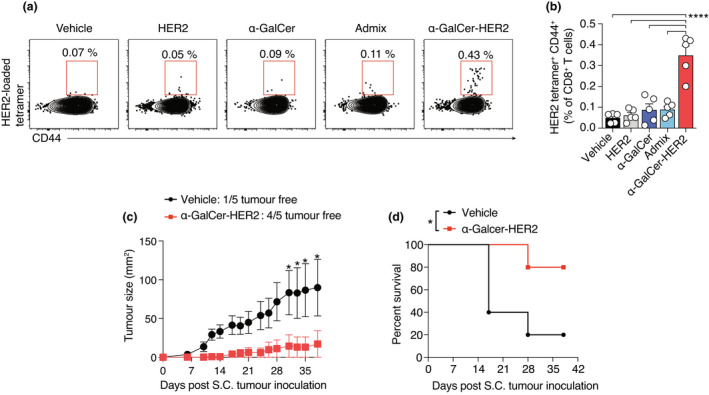
A single dose of α‐GalCer‐HER2 primes HER2‐specific CD8^+^ T cells and delays subcutaneous tumor outgrowth. **(a)** Typical flow cytometry plots displaying staining with H‐2K^d^/HER2_63‐71_ tetramer in peripheral blood of mice immunised with 20 nmol of either α‐GalCer‐HER2, α‐GalCer or PBS vehicle 7 days earlier. **(b)** Frequency of HER2‐specific CD8^+^ T cells in peripheral blood of mice. The percentage of HER2 tetramer^+^ CD44^+^ CD8^+^ T cells is shown as mean ± SEM with five mice per group. The results of one of three independent experiments are shown. **(c)** Mice were challenged with 1 × 10^5^ 4T1.2‐HER2 tumor cells S.C. 7 days after I.V. vaccination with 20 nmol α‐GalCer‐HER2 or mock vaccination with vehicle. Tumor growth curves for each mouse, with 5 mice per group. **(d)** Kaplan–Meier curves showing the percentage survival to endpoint. *P < 0.05, ****P < 0.0001; **(b)** one‐way ANOVA with Tukey's multiple comparison test; **(c)** two‐way ANOVA with Tukey's multiple comparison test; **(d)** Gehan‐Breslow‐Wilcoxon test.

To assess the capacity of the conjugate vaccine to enhance peptide‐specific T‐cell responses, BALB/cJ mice were vaccinated with either α‐GalCer‐HER2, an admix of unconjugated α‐GalCer and HER2 peptide or each component alone (all at equimolar concentrations). The frequency of HER2‐specific CD8^+^ T cells in the peripheral blood was determined 7 days after vaccination by flow cytometry using HER2_63−__71_‐loaded H‐2K^d^ tetramers. Only the conjugate vaccine induced increases in HER2 tetramer‐positive CD8^+^ T cells that were significantly increased over animals injected with vehicle alone (Figure 3a and b). Antitumor responses were then assessed in animals that were injected with 4T1.2‐HER2 tumor cells subcutaneously 7 days after vaccination. Tumor growth was delayed in α‐GalCer‐HER2‐vaccinated mice, with a significant survival advantage (Figure 3c and d).

### Glycolipid‐conjugate vaccine prevents 4T1.2‐HER2 colonisation of the lung

**Figure 4:**
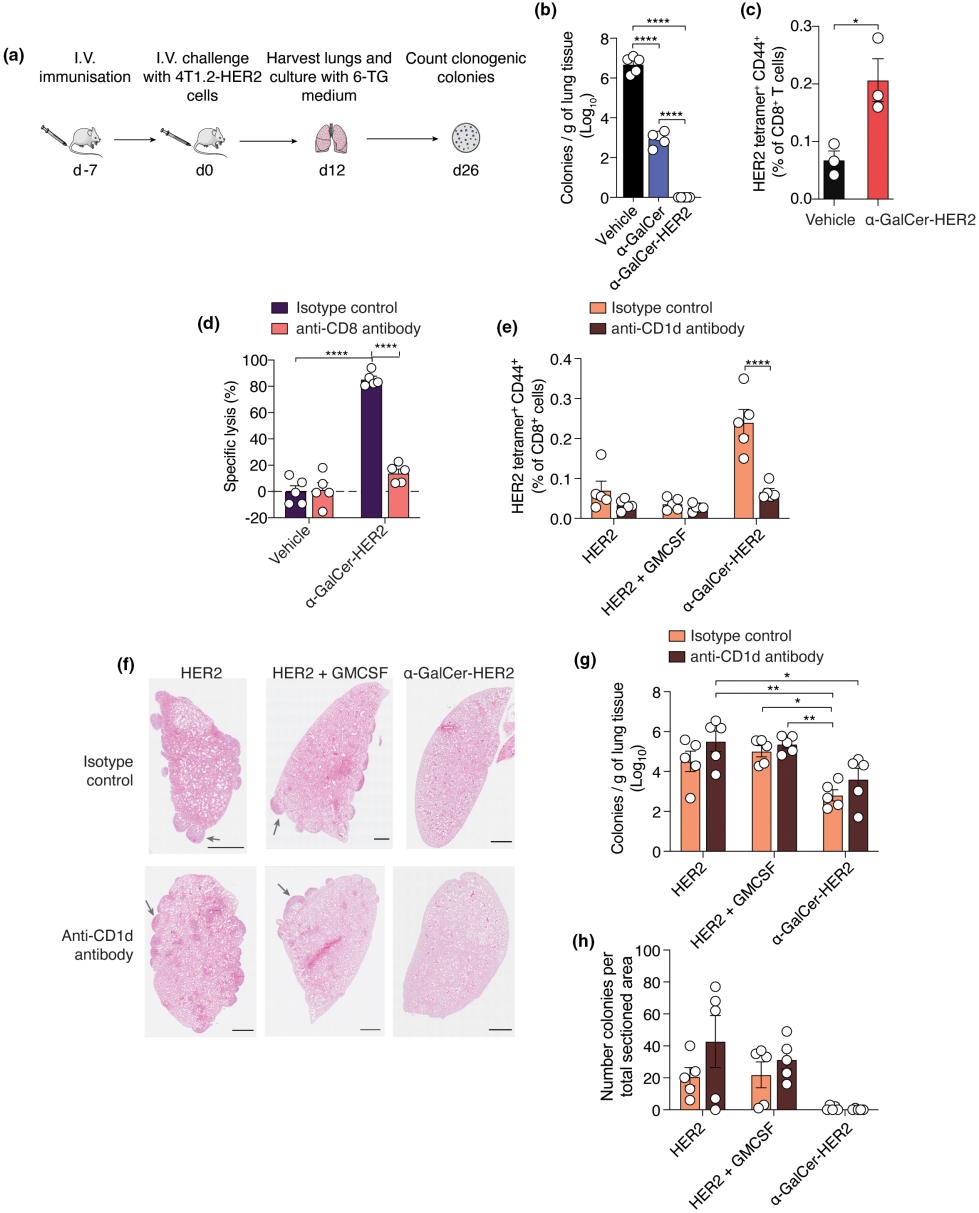
A glycolipid‐HER2 peptide conjugate vaccine prevents lung colonisation by 4T1.2‐HER2 cells more effectively than GM‐CSF. **(a)** Schematic of the experimental protocol. Mice were immunised with 20 nmol α‐GalCer‐HER2 or α‐GalCer 7 days before I.V. challenge with 5 × 10^5^ 4T1.2‐HER2 tumor cells. Mice were sacrificed on day 12 after tumor challenge, and the presence of tumor lesions in lung assessed by 6‐TG clonogenic assay. **(b)** Mean number of colonies per gram of lung tissue for each treatment group (*n* = 5). **(c)** Frequency of HER2‐specific CD8^+^ T cells in the lungs of mice ± SEM (*n* = 3). **(d)** BALB/cJ mice were immunised I.V. with 20 nmol α‐GalCer‐HER2 or vehicle. Anti‐CD8 depleting antibody or IgG control antibody was administered I.P. at 200 μg mouse^−1^ on days 5 and 6 after immunisation. Mice were challenged with HER2_63‐71_‐pulsed syngeneic splenocytes on day 7 and the cytotoxic T‐cell response against these assessed the following day in peripheral blood. **(e)** Frequency of HER2‐specific CD8^+^ T cells in the spleens of mice upon sacrifice 12 days after tumor cell challenge. Same experimental design as in **(a)** except mice were administered isotype control antibody or anti‐CD1d antibody I.P one day prior to vaccination. GM‐CSF was administered I.V. at 5 μg per mouse alongside 20 nmol HER2 peptide to the indicated groups at day 0. **(f)** H&E staining of lung lobe. Representative image from each treatment group shown. Arrows indicate examples of tumor nodules. **(g)** Mean number of colonies per gram of lung tissue for each treatment group as detected *via* clonogenic assay (*n* = 5). **(h)** Total number of colonies visible in the four lung sections taken from each mouse. Shown is the number for each mouse per treatment group with *n* = 5. *P < 0.05; **P < 0.01 ****P < 0.0001; **(b, d, e, g, h)** one‐way ANOVA with Tukey's multiple comparison test; **(c)** Mann–Whitney *U*‐test.

Clinically, antitumor vaccination is most likely to be employed in the adjuvant setting, with the goal of preventing distant metastasis, including to the lung, after treatment of the primary tumor. Therefore, we assessed the capacity of vaccination to prevent lung colonisation in the 4T1.2‐HER2 breast cancer model. BALB/cJ mice received the α‐GalCer‐HER2 vaccine, α‐GalCer or vehicle control 7 days before intravenous challenge with 4T1.2‐HER2 tumor cells. Mice were euthanised 12 days after tumor challenge, and the presence of lung tumors assessed by enumerating colonies formed after exposing harvested lung tissue to 6‐TG, a chemotherapeutic agent to which 4T1 cells are intrinsically resistant (Figure 4a). As anticipated, a large number of colony‐forming units (CFUs) were observed in the lungs of control mice (Figure 4b). A significant reduction in CFUs was observed in α‐GalCer‐treated mice, indicating α‐GalCer alone partially suppresses lung colonisation by 4T1.2‐HER2 cells. However, mice pretreated with an equimolar concentration of the α‐GalCer‐HER2 vaccine had no tumor colonies in the lungs, consistent with a protective effect of the antigen‐specific T‐cell response. In a separate experiment, HER2‐specific CD8^+^ T cells were detected in the lungs of mice 19 days after vaccination with α‐GalCer‐HER2 (Figure 4c).

To determine the function of the vaccine‐generated CD8^+^ T cells, their ability to induce cytotoxicity against HER2_63‐71_ peptide‐loaded targets was assessed *in vivo* seven days after vaccine administration (Figure 4d). A strong cytotoxic response resulting in elimination of target cells in peripheral blood was observed following α‐GalCer‐HER2 treatment (Figure 4d). When CD8^+^ cells were depleted from hosts after vaccination, this cytotoxicity was abrogated, suggesting that it was mediated by cytotoxic CD8^+^ T cells (Figure 4d). Whilst NKT cells are likely involved in the initial priming of HER2^+^ CD8^+^ T cells through their cellular adjuvant activity, these data suggest the peptide‐specific cytotoxicity against HER2‐pulsed splenocytes is not attributable to NKT cells alone, which are predominantly CD8 negative.^37^ These mice were further challenged with 4T1.2‐HER2 tumor cells intravenously 7 days after the cytotoxicity assay. Assessment of tumor cell colonisation of the lung 12 days later revealed a decrease in tumor metastases in the α‐GalCer‐HER2‐vaccinated group with no significant difference to those mice also administered the anti‐CD8 depleting antibody (Supplementary figure 5a). These results suggested the vaccine‐activated NKT cells may play an additional role in suppressing tumor growth. As anticipated, blocking CD1d and thus the ability of α‐GalCer to activate NKT cells had a significant effect on the HER2‐specific CD8^+^ T‐cell response in comparison with those that received the isotype control (Figure 4e). Furthermore, vaccination of mice with HER2 peptide alone or in combination with GM‐CSF, a common adjuvant for breast cancer peptide vaccines in the clinic, failed to induce strong HER2‐specific responses (Figure 4e) or the increases in serum cytokines that are seen in response to the α‐GalCer‐HER2 vaccine (Supplementary figure 5b). HER2 peptide alone or in combination with GM‐CSF also failed to induce a strong antitumor effect, with a large number of CFU observed in the lungs of these mice, as assessed by both lung histology, and using a clonogenic assay (Figure 4f, g and h). A significant reduction in CFUs was observed in the α‐GalCer‐HER2 vaccine co‐administered the isotype control (Figure 4g). In this assay, the level of CFUs was similar in α‐GalCer‐HER2‐treated mice, whether treated with anti‐CD1d or isotype control (Figure 4g). Similar results were obtained when tumors were quantified as the percentage area of the lung (Supplementary figure 5b).

### NY‐ESO‐1‐targeting glycolipid‐conjugate vaccine prevents metastatic spread to lungs

HER2‐directed therapy is not an option for TNBC, which carries a poor prognosis and presents a high clinical need. TNBC, however, frequently over‐expresses the cancer‐testis antigen NY‐ESO‐1.^28^ Therefore, we designed and synthesised a third glycolipid‐peptide conjugate vaccine directed against the NY‐ESO‐1 antigen (α‐GalCer‐NY‐ESO‐1) which contains the H‐2D^d^‐binding CD8^+^ epitope NY‐ESO‐1_81–88_ and can therefore be assessed in BALB/cJ mice.

**Figure 5:**
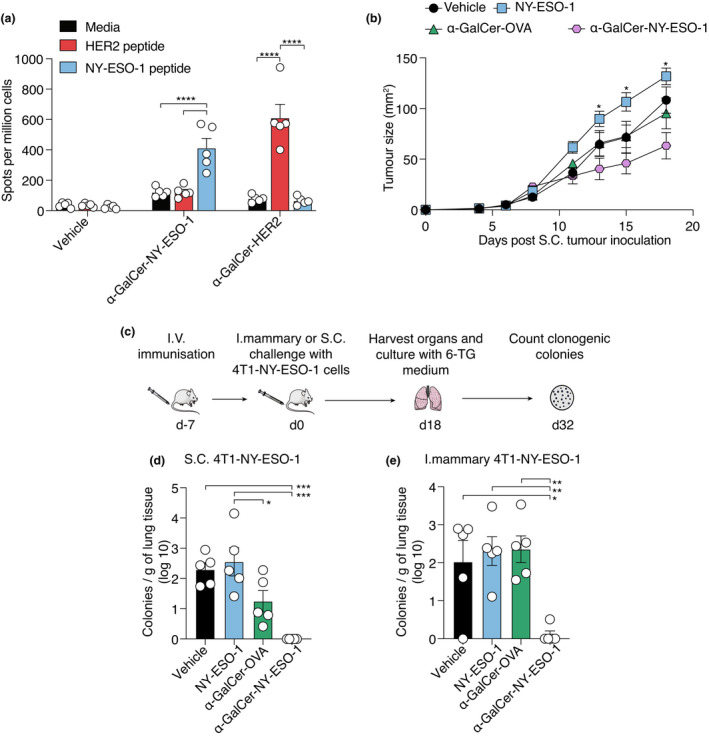
A glycolipid‐NY‐ESO‐1 peptide conjugate vaccine elicits NY‐ESO‐1‐specific T‐cell responses and reduces metastasis of 4T1‐NY‐ESO‐1 cells. **(a)** Mean ± SEM of NY‐ESO‐1‐specific or HER2‐specific IFN‐γ producing splenocytes quantified by ELISpot assay seven days after administration of 3 nmol α‐GalCer‐NY‐ESO‐1 or α‐GalCer‐HER2. (**b**) Mice were vaccinated with 3 nmol of either NY‐ESO‐1_177‐122_ peptide alone (NY‐ESO‐1), the α‐GalCer‐NY‐ESO‐1 vaccine, a vaccine incorporating an irrelevant antigen (α‐GalCer‐OVA) or vehicle 7 days before 4T1‐NY‐ESO‐1 tumor cells were injected by the subcutaneous (1 × 10^5^) route. Tumor growth plots showing mean tumor volume ± SEM; 5 animals per group, representative of two similar experiments.   **(c)** Schematic of experimental design. Mice were vaccinated with 3 nmol of either NY‐ESO‐1 peptide alone, the α‐GalCer‐NY‐ESO‐1 vaccine, a vaccine incorporating an irrelevant antigen (α‐GalCer‐OVA) or vehicle 7 days before 4T1‐NY‐ESO‐1 tumor cells were injected by either the subcutaneous (1 × 10^5^) or intra‐mammary (5 × 10^4^) route. At day 18 lungs tissues were collected and assessed for the presence of metastatic tumors by 6‐TG colony‐forming assay. **(d)** Mean number of colonies per gram of lung tissue ± SEM for each of the treatment groups in animals with subcutaneous tumor challenge, or **(e)** tumor injected into the mammary fat pad. Representative of two similar experiments. *P < 0.05, **P < 0.01, ***P < 0.001, ****P < 0.0001; **(a, b)** two‐way ANOVA with Tukey's multiple comparison test; **(d, e)** one‐way ANOVA with Tukey's multiple comparison test.

We first assessed the vaccine‐generated T‐cell response by IFN‐γ ELISpot. Stimulation of splenocytes from α‐GalCer‐NY‐ESO‐1‐vaccinated mice with the NY‐ESO‐1_81–88_ peptide *in vitro* elicited an increased number of IFN‐γ‐producing T cells relative to splenocytes cultured in medium only (Figure 5a). No such response was seen with the irrelevant HER2_63‐71_ peptide. Correspondingly, splenocytes from α‐GalCer‐HER2‐vaccinated mice did not respond to stimulation with NY‐ESO‐1_81–88_ (Figure 5a).

As had been seen with vaccination in the previous 4T1 model, subcutaneous tumor growth of 4T1 cells expressing NY‐ESO‐1 was delayed in α‐GalCer‐NY‐ESO‐1‐vaccinated mice in comparison with vehicle‐treated mice (Figure 5b). Because we found that this particular tumor model spontaneously metastasises more readily than 4T1‐HER2, we were able to assess the capacity of the vaccine to prevent metastatic spread from initial tumor lesions implanted subcutaneously or orthotopically in the mammary tissue. Thus, seven days after vaccination, mice were injected with 4T1‐NY‐ESO‐1 cells, and 18 days later, the lungs were assessed for tumor growth using the 6‐TG colony‐forming assay (Figure 5c). A significant reduction in the number of lung colonies was observed in mice that has received α‐GalCer‐NY‐ESO‐1 in comparison with those vaccinated with NY‐ESO‐1 peptide alone or vehicle control in both the subcutaneous (Figure 5d) and the orthotopic (Figure 5e) models. In the subcutaneous model, an irrelevant vaccine (α‐GalCer‐OVA) did limit the number of colonies, but not to the extent achieved with the vaccine, implying some NKT cell‐mediated antitumor activity is involved.

## Discussion

Here, we report that a single dose of an NKT cell‐activating glycolipid‐peptide conjugate vaccine can generate cytotoxic CD8^+^ T‐cell responses, delay tumor growth *in vivo* and prevent tumor colonisation of the lung in models of breast cancer metastasis.

This study is the first to investigate NKT cell‐activating glycolipid‐peptide conjugate vaccines to prevent breast cancer and related metastasis to the lungs. We have demonstrated *in vivo* antitumor activity using two established breast cancer cell lines and three different antigens. Through tetramer staining, we demonstrated activation and expansion of both NKT and peptide‐specific CD8^+^ T cells, and *via* the use of glycolipid‐only, irrelevant‐peptide controls, and depletion of CD8^+^ T cells, we show this tumor protection is peptide‐specific. We demonstrate that chemical conjugation of peptide and adjuvant results in greater antitumor responses than when the two are co‐administered, a phenomena also reported for nanoparticle‐based vaccines in a HER2 breast cancer model.^38^ Compared with the combination of GM‐CSF with HER2 peptide, employing a concentration of GM‐CSF reported to enhance antitumor and antiviral immune responses when co‐administered with peptides,^39^ the α‐GalCer‐HER2 vaccines induced a significantly greater peptide‐specific response and antitumor response. Furthermore, we observe efficacy after a single vaccine dose, and unlike some vaccine strategies that exploit NKT cells, such as administration of irradiated tumor cells pulsed with α‐GalCer,^40^ or autologous α‐GalCer‐pulsed dendritic cells,^41^, ^42^ the synthetic glycolipid‐peptide conjugate vaccines we employ avoid the need for personalised cellular product manufacture. Finally, our research employed two vaccine conjugates that target clinically relevant antigens: human HLA‐A*2402‐positive individuals possess CD8^+^ T cells that are reactive to the same HER2_63‐71_ peptide^43^ used within one of our conjugates, and in TNBC, where the need for new adjuvant therapies may be greatest,^44^ NY‐ESO‐1 has been identified as a potential target antigen.^28^ In the case of NY‐ESO‐1, the evaluated vaccine incorporated a long peptide that encompasses many of the known epitopes expressed in humans.^45^, ^46^ Conjugate vaccines incorporating long peptides such as this offer the potential to incorporate the most immunogenic regions of a tumor antigen, giving a high likelihood of inducing responses in any individual despite high MHC diversity in the human population.^41^

Our work has limitations. Subcutaneous and intravenous tumor cell administration as we used for the 4T1.2‐HER2 cell line are simplified models of metastasis. We used these models to overcome local ulceration observed following orthotopic (intramammary) implantation of 4T1.2‐HER2 cells, and occasional spontaneous regression seen with this cell line in long‐term experiments in wild‐type mice, a phenomenon that has previously reported.^47^ For this reason, we confirmed our findings in a spontaneous metastasis model using an NY‐ESO‐1‐expressing 4T1 cell line implanted orthotopically, which demonstrated more consistent growth kinetics in our hands. Although we report robust protection against lung tumor colonies, we did not observe complete rejection of subcutaneously‐ or intramammary‐implanted tumors. This may be due to the immunosuppressive tumor stroma at primary tumor sites and is consistent with reports that cancer vaccine monotherapy fails to eliminate established tumors in other breast cancer models.^48^, ^49^, ^50^ While it is possible that additional strategies, such as combining the vaccines with checkpoint blockade therapies or cytotoxic agents, might have improved control of the primary tumor, cancer vaccines are more likely to be used as adjuvant therapies to prevent metastasis, a role which our findings support.

Humans have a lower frequency of NKT cells than mice, raising the question of whether NKT cell‐based vaccine adjuvants will translate to the clinic. Reassuringly, we demonstrate vaccine efficacy in BALB/cJ mice, which have a smaller and more Th2‐skewed NKT cell population than the commonly employed C57Bl/6J mouse strain.^24^ Moreover, we have previously shown that similar glycolipid‐peptide conjugate vaccines can stimulate antiviral CD8^+^ T‐cell responses within human peripheral blood mononuclear cells,^22^ and a human randomised clinical trial reported that an NKT cell‐activating glycolipid enhanced antibody responses against a poorly immunogenic protein antigen.^51^ Together, these findings suggest that the low NKT cell frequency observed in humans is not a barrier to the efficacy of NKT cell‐stimulating vaccine adjuvants.

Traditional vaccine approaches aim to stimulate both CD4^+^ and CD8^+^ T cells, in part because CD4^+^ T‐cell help can enhance cytotoxic CD8^+^ T‐cell responses. For example, Brown *et al*.^52^ administered the CD4^+^ T cell‐activating peptide li‐Key/HER2_776–790_ (AE37) alongside the CD8^+^ T cell‐activating peptide HER2_654–662_ (GP2), hypothesising that the former would provide help to enhance responses against the latter. Like conventional CD4^+^ T‐cell help, CD1d‐restricted NKT cell‐activating glycolipids can provide NKT cell ‘help’ for CD8^+^ T‐cell and antibody responses, but do so in a manner independent of MHC Class II.^16^, ^17^, ^18^, ^53^, ^54^, ^55^, ^56^ Our findings using glycolipid‐peptide conjugate vaccines are consistent with NKT cell‐dependent help for cytotoxic CD8^+^ T‐cell responses, as we show here for the OVA conjugate through the use of CD1d knockout animals. However, some of the enhanced activity we observe upon conjugating glycolipid to an antigenic peptide may relate to altered pharmacokinetic or pharmacodynamic characteristics of the peptide following its lipidation.^57^ This may explain the failure of an anti‐CD1d antibody to abrogate vaccine‐induced antitumor responses; alternatively, the CD1d blockade we employed may have been transient or incomplete. We observed a reduction in lung metastases with α‐GalCer alone, consistent with a direct antitumor effect of NKT cells, or with NKT‐cell induced enhancement of innate immunity.^58^, ^59^, ^60^, ^61^ Indeed, administration of high dose α‐GalCer has been shown to prevent lung metastases in models of 4T1 breast cancer, and to result in increases in NK cell IFN‐γ production.^62^ Our data are consistent with the concept that activated NKT cells provide an antitumor response that is not peptide‐specific. However, a greater antitumor response was induced by the conjugate vaccine in a peptide‐relevant context, as opposed to an irrelevant peptide setting, suggesting peptide‐specific T cells were contributing as well.

The breast cancer vaccine nelipepimut‐S employs the cytokine GM‐CSF as an adjuvant, which matures dendritic cells, but has the potential to stimulate myeloid‐derived suppressor cells, paradoxically suppressing cellular immunity.^63^ Ligands for TLRs are frequently used as cancer vaccine adjuvants,^64^ and like NKT cell‐activating glycolipids, can be conjugated to antigenic peptides.^65^ The mechanism of NKT cell‐activating glycolipids as vaccine adjuvants, however, differs from that of both GM‐CSF and TLR ligands. Similarly to traditional CD4^+^ T‐cell help, NKT cell‐based adjuvants elicit CD40/CD40L‐dependent NKT cell licensing of antigen‐presenting cells independently of the TLR adaptor protein MyD88.^14^, ^17^ Notably, TLR ligands can cooperate with NKT cell ligands to further enhance peptide‐specific T‐cell responses and combinations could be assessed.^66^ Furthermore, as this treatment is likely to be most effective in an adjuvant setting, combination of glycolipid‐peptide vaccines alongside standard treatment options, such as chemotherapy, may be necessary; encouragingly, the oncolytic peptide LTX‐315 was found to enhance the antitumor effect of doxorubicin in the 4T1 model.^67^

Although peptide vaccines elicit measurable immune responses in people with breast cancer, clinically meaningful disease‐free survival benefits have not yet been demonstrated.^68^, ^69^ This may relate to selection of peptide antigen, an inability to overcome tumor‐related immunosuppression or choice of vaccine adjuvant. In the current study, we generated conjugate vaccines based on HER2 and NY‐ESO‐1 peptides. While both are accepted antigenic targets in breast cancer, neither is yet proven in terms of clinical response to peptide vaccination. Advances in gene sequencing and bioinformatics have opened the door to personalised peptide cancer vaccines based on patient‐specific neoantigens consisting of multiple antigens.^70^ This strategy is of particular interest for TNBC, which typically carries a high mutational burden.^71^ To generate the conjugate vaccines, we used aniline catalysed oxime ligation to ‘click’ antigenic peptides *via* an immolative linker to adjuvant compounds.^72^ This method produces clean reaction products requiring minimal purification, so is amenable for the simultaneous production of multiantigen products, and represents a potential avenue to the synthesis of personalised conjugate vaccines to Good Manufacturing Practice standards.^73^

## Conclusions

A class of glycolipid‐peptide conjugate vaccines activate both NKT cells and peptide‐specific CD8^+^ T cells and prevent tumor cell spread to the lungs in models of breast cancer metastasis. Glycolipid‐peptide conjugate vaccines warrant investigation as an adjuvant strategy to prevent metastasis of high‐risk breast cancer.

## Methods

### Mice

All mice were bred and housed in the Biomedical Research Unit of the Malaghan Institute of Medical Research, Wellington, New Zealand. Experimental protocols were approved by the Victoria University Animal Ethics Committee (protocol reference: 23784 and 26384) and performed according to institutional guidelines. Age‐ and sex‐matched mice between 6 and 12 weeks of age were used. Mice were maintained on meat‐free rat and mouse rodent chow (Specialty Feeds, Western Australia) and acidified water *ad libitum*, with a 12‐h light/dark cycle. Male and female mice from the following strains were used: C57BL/6J (originally from Jackson Laboratories, Bar Harbour, ME, USA); CD1d^−/−^ mice,^74^ which are devoid of CD1d‐restricted NKT cells; BALB/cJ mice (Jackson Laboratories).

### Cell lines, medium and reagents

Cell lines were maintained in aseptic conditions through use of Class II biological safety cabinets with HEPA air filters (HERAsafe, Heraeus, Germany). Cells were incubated in humidified incubators at 37 °C with 5% CO_2_ (HERAcell incubator, Heraeus, Germany). All cell lines were found to be free of contamination by mycoplasma *via* the MycoAlert™ mycoplasma detection kit (Lonza, Walkersville, USA).

The ovalbumin (OVA)‐expressing E0771 breast cancer cell line was gifted from Dr Paul Beavis (Peter MacCallum Cancer Centre, Melbourne, Australia). The cells were cultured in Dulbecco's modified eagle's medium (DMEM) (Gibco, Grand Island, NY, USA) supplemented with 10% foetal bovine serum (FBS) and 2 mm glutamax (both Gibco). The 4T1.2‐HER2 cell line was gifted by Professor Phillip Darcy (Peter MacCallum Cancer Centre, Melbourne, Australia) and expresses the human HER2 protein and green fluorescent protein (GFP).^34^ Cells were grown in Roswell Park Memorial Institute (RPMI) 1640 medium supplemented with 10% FBS, 100 U mL^−1^ penicillin, 100 μg mL^−1^ streptomycin, 55 μM 2‐ME (termed cRPMI; all Gibco). The 4T1.2‐HER2 cell line is a subclone of the original 4T.1 epithelial tumor cell line derived from the mammary gland of a BALB/c *f*. C3H mouse that has been reported to depict stage IV human breast cancer due to its highly metastatic potential.^75^ The 4T1‐NY‐ESO‐1 GFP^+^ cell line was gifted by Dr Uzi Gileadi, Oxford, England. To ensure the expression of NY‐ESO‐1 and GFP on the cells and optimal growth in our BALB/cJ mouse cohort, a cell line was generated in our facility from a single 4T1‐NY‐ESO‐1 GFP^+^ cell. Tumors established from this cell line were harvested and processed *ex vivo* in medium containing the cytotoxin, 6‐thioguanine (6‐TG; Sigma‐Aldrich, MO, USA). As all 4T1‐derived strains are resistant to 6‐TG‐induced cell death, this selected for a pure population of 4T1‐NY‐ESO‐1 tumor cells in culture. These cells were then harvested, sorted for CD45^−^ GFP^+^ cells, aliquoted, and frozen in lots of 5 × 10^6^ cells. Cells cultured from these frozen stocks were used for all 4T1‐NY‐ESO‐1 experiments, where, one day before tumor cell challenge, the cells were sorted once more for GFP^+^ cells, cultured overnight and then harvested for injection.

Recombinant murine GM‐CSF (Peprotech, NJ, USA) was administered intravenously at 5 μg per dose, a concentration observed by others to induce antitumor and antiviral immune responses in mice.^39^, ^76^, ^77^, ^78^

### Breast tumor model

For S.C. tumor cell challenge, tumor cells were harvested, processed through a 70‐μm filter, and resuspended in RPMI medium for injection. Mice were injected unilaterally into the flank with 5 × 10^5^ E0771‐OVA tumor cells, or 1 × 10^5^ cells for both the 4T1.2‐HER2 cells 4T1‐NY‐ESO‐1 tumor cell lines. For intramammary (I.mammary) challenge, 1 × 10^4^ 4T1.2‐HER2 tumor cells or 5 × 10^4^ 4T1‐NY‐ESO‐1 tumor cells were injected in 20 μL of RPMI medium into the 2nd mammary fat pad. Tumor growth was monitored every 2–3 days, with tumor size calculated as the product of the two bisecting diameters. Mice were euthanised when subcutaneous tumors reached > 200 mm^2^ or intramammary tumors reached > 50 mm^2^ for the HER2 model and > 120 mm^2^ in the NY‐ESO‐1 model.

For intravenous (I.V.) tumor challenge, 5 × 10^5^ 4T1.2‐HER2 tumor cells were administered *via* the lateral tail vein in 200 μL of RPMI medium.

### Preparation of α‐GalCer and synthetic vaccines

The NKT cell agonist α‐GalCer was manufactured in‐house according to the published procedure.^79^ Solubilisation was achieved by freeze‐drying in the presence of sucrose, L‐histidine, and Tween 20,^80^ and resuspending in sterile injection water to give a stock concentration of 582 μM; this was further diluted in PBS to give the required dose for I.V. injection.

The glycolipid‐peptide conjugate vaccines consisted of a prodrug form of α‐GalCer, which readily reverts to a more stable *N*‐acyl form under physiological conditions following cleavage of a traceless linker by cathepsin B.^20^ The sequence FFRK was added to the N‐terminus of the peptides to promote proteolytic release of the minimal MHC‐binding peptide.^81^ Vaccines were synthesised as previously reported,^21^ conjugating peptides that were N‐terminally modified with aminooxyacetic acid (AoAA) for oxime ligation with the ketone group of the pro‐adjuvant. Thus, for targeting OVA, a vaccine was prepared with AoAA‐FFRK‐KISQAVHAAHAEINEAGRESIINFEKLTEWT, which is a non‐contiguous sequence from OVA containing the I‐A^b^‐restricted CD4 epitope OVA_323–339_ (KISQAVHAAHAEINEAGR) fused to the H‐2K^b^‐restricted CD8^+^ T‐cell epitope OVA_257–264_ (SIINFEKL). The final product is referred to as α‐GalCer‐OVA. For targeting human HER2, the peptide was AoAA‐FFRK‐TYLPTNASL, containing an H2‐K^d^ and HLA‐A2402‐restricted epitope HER2_63‐71_ (TYLPTNASL), with the vaccine referred to as α‐GalCer‐HER2. For targeting NY‐ESO‐1, a vaccine was prepared with AoAA‐FFRK‐GARGPESRLLEFYLAMPFATPMEAELARRSLAQDAPPL, a long contiguous sequence from the protein that encompassed the H‐2D^d^‐binding epitope NY‐ESO‐1_81–88_ (RGPESRLL), and is referred to as α‐GalCer‐NY‐ESO‐1. All peptides were prepared in‐house. The vaccines were typically lyophilised in the presence of solubilisation matrix, as for α‐GalCer above, and resuspended to 0.5 mg mL^−1^ in sterile injection water, and diluted in PBS to the desired concentration for I.V. injection (3 nmol mouse^−1^). However, α‐GalCer‐HER2 was dissolved in DMSO to 10 mg mL^−1^and diluted in PBS to give the desired concentration of 100 μM for I.V. injection (20 nmol mouse^−1^). The synthesis of peptides and characterisation of conjugate vaccines is described in the Supplementary Information.

### Vaccine administration

Vaccines were administered I.V. *via* the lateral teil vein. Mice were first warmed under a heat lamp before being restrained and 200 μL administered in a slow continuous stream using an Ultra‐Fine 1‐mL 27‐gauge syringe (BD; New Jersey, USA).

In some experiments, CD8^+^ cell depletion was achieved using intraperitoneal injection of 200 μg anti‐CD8 depleting antibody (clone 2.43; BioxCell, New Hampshire, USA) on day 5 and 6 post‐vaccination. An additional group of mice was administered an isotype control antibody (rat IgG2b). Depletion was checked in the peripheral blood on day 7 post‐vaccination (Supplementary figure 4).

### Cytokine analysis

Blood was collected *via* cheek bleeding (submandibular vein puncture) into a 1.7 mL microcentrifuge tube and left to rest at room temperature (RT) for 3 h. Following this, the tubes were spun at 21130 *g* for 10 min and the serum layer collected into a new tube. Analysis of serum cytokine levels was performed using the Bio‐Plex Pro Mouse Cytokine 23‐plex assay following the manufacturer's instructions (Bio‐Rad) and measured using the Luminex® 200™ multiplexing instrument. The values were normalised to control (PBS)‐treated mice, transformed by taking the base‐2 logarithm, and displayed in a heat map with red indicating expression greater than PBS treated mice and blue representing lower expression. As there was insufficient serum to repeat the assay, samples with cytokine values below the limit of detection were ascribed to the lowest concentration that was revealed across the samples for that particular cytokine, as this has been reported to be more accurate than omitting these samples or replacing them with a value of zero.^82^

### Flow cytometry analysis

Flow cytometry was used to assess the effect of the vaccines on the activity of NKT cells and APCs. To assess NKT cells, spleens were harvested seven days after vaccine administration. For the effect on APCs, spleens were harvested at 18 h post‐vaccine administration, processed through a 70‐μm filter with Iscove's Modified Dulbecco's Medium (IMDM) and centrifuged at 572 *g* for 4 min. Red blood cells (RBC) were lysed with RBC lysis solution (QIAGEN, Valencia, CA, USA). Following this, samples were washed by centrifugation and the pellet resuspended in FACs buffer (PBS supplemented with 1% FBS, 0.01% NaN_3_, 2 mm EDTA). One‐tenth of the spleen sample was plated in a 96‐well U‐bottom shaped plate for staining. Cells were first incubated with the viability dye NIR Zombie™ (1:1000; BioLegend) for 15 min at RT. Samples were then washed by centrifugation at 800 *g* for 2 min in FACs buffer and incubated with Fc‐block (24G2; 1:100 in‐house synthesis) for 10 min at 4 °C. The wash step by centrifugation was repeated. For assessment of NKT cells, samples were incubated for 15 min at RT in the dark with 0.5 μL of PBS‐57‐loaded CD1d tetramer, with PBS‐57 being an analogue of α‐GalCer (NIH tetramer Core facility, Emory University). Antibody mixtures containing the desired antibodies diluted in FACs buffer to the predetermined optimal concentrations were added in 50 μL volumes to each well for 20 min (optimised flow cytometry panels provided in Supplementary tables 1 and 2); the cells were then washed twice and resuspended in FACs buffer. Samples were analysed on a 3‐laser spectral flow cytometer Aurora (Cytek Biosciences) using SpectroFlo software version 2.2. The data were analysed using FlowJo v10.5.3 software, with the gating strategy for NKT cell and APC analysis shown in Supplementary figures 1 and 2, respectively.

### *In vivo* cytotoxicity assay

The cytotoxic capacity of vaccine‐induced CD8^+^ T‐cell responses was measured by VITAL assay as previously described.^83^ As targets, syngeneic splenocyte populations were loaded with one of three concentrations of the minimal HER2_63‐71_ peptide (10 μM, 1 μM, 0.1 μM; GenScript, NJ, USA). An additional population of cells was left unloaded. Cells loaded with antigen were then labelled with one of three concentrations of the fluorescent dye carboxyfluorescein succinimidyl ester (CFSE; Thermofisher, 5 mM; 1 mM; 0.2 mM). Cells without antigen were labelled with chloromethyl‐benzoyl‐aminotetramethyl‐rhodamine (CMTMR; 10 μM; both Molecular Probes, Eugene, OR, USA). Labelling with CFSE was in PBS (2 × 10^6^ cells mL^−1^) supplemented with 333 nm CFSE at room temperature for 8 min, followed by the addition of an equal volume of FBS to quench the reaction. The cells were then washed twice and resuspended in IMDM for counting. Labelling with CMTMR was in complete medium (2 × 10^6^ cells mL^−1^) supplemented with 10 μM CMTMR at 37 °C for 15 min followed by incubation in fresh complete medium for a further 15 min at 37 °C. Following two washes, cells were resuspended in IMDM for counting. Equal proportions of each cell population were mixed together and injected I.V. into groups of vaccinated or naïve mice (*n* = 3–5; each mouse received 6 × 10^6^ cells in total). Specific lysis of the peptide‐loaded targets was assessed 24 h later in the peripheral blood by flow cytometry. Mean percentage of survival of peptide‐pulsed targets was calculated relative to that of the control population, and cytotoxic activity was expressed as a percentage of specific lysis using the calculation: (100 − mean percentage of survival of peptide‐pulsed targets).

### Monitoring CD8 ^+^ T‐cell responses *ex vivo* with MHC class I/peptide tetramers

Peripheral blood samples were collected *via* the submandibular vein into 200 μL of 0.5 mm ethylenediaminetetraacetic acid (EDTA; Invitrogen) in PBS, centrifuged at 1500 *g* for 4 min and the supernatant removed. For analysis of HER2‐positive cells in the lungs, samples were harvested on day 18 post‐vaccination, cut into small pieces and incubated in a digestion mix of Liberase TL and DNase I (both Roche, Mannheim, Germany) for 45 min at 37 °C, processed through a 70‐μm filter and centrifuged at 572 *g* for 4 min. For all samples, RBCs were lysed with RBC lysis solution, centrifugation was repeated and the pellet resuspended in FACs buffer, and plated in a 96‐well plate for staining. Cells were first incubated with the viability dye, NIR Zombie (1:1000) for 15 min at RT. Cells were then washed by centrifugation at 800 *g* for 2 min in FACs buffer and incubated with Fc‐block (24G2; 1:100) for 10 min at 4 °C. The wash step was repeated before incubation with the multimer complex that corresponded to the peptide of interest for 15 min at RT in the dark. These included the H‐2K^b^/OVA_257–264_ pentamer complex (ProImmune, Oxford, UK) and the H‐2K^d^/HER2_63–71_ tetramer complex (NIH tetramer Core facility, Emory University). Antibody mixtures containing the desired antibodies (see Supplementary table 3) diluted in FACs buffer to the predetermined optimal concentrations were added in 50 μL volumes to each well for 20 min; the cells were then washed twice and resuspended in FACs buffer. Samples were analysed on a 3‐laser spectral flow cytometer Aurora (Cytek Biosciences) using SpectroFlo software version 2.2. The data were analysed using FlowJo v10.5.3 software, with the gating strategy provided in Supplementary figure 3.

### IFN‐γ ELISpot assay

Mouse IFN‐γ ELISpot^PLUS^ kits from Mabtech were used to measure the number of T cells engaged in IFN‐γ production following antigen restimulation. The assay was performed following the manufacturer's recommendations. Briefly, on day 7 post‐vaccination, spleens were collected from euthanised mice and processed to a single cell suspension. Splenocytes at 3 × 10^5^ per well were added to a 96‐well ELISpot plates precoated with anti‐IFN‐γ. These splenocytes were stimulated with 10 μM of the minimal peptides for each of the vaccines of interest, *for instance* the HER_63‐71_ peptide and NY‐ESO‐1_81–88_ peptide. Control wells were incubated with cRPMI medium only. Following incubation at 37 °C + 5% CO_2_ for 18 h, cells were discarded and wells were washed five times with PBS. Biotinylated detection antibody diluted in 0.05% FBS/PBS was added to wells and incubated at RT for 2 h and washed as above. Wells were then incubated with streptavidin‐alkaline phosphatase diluted in 0.05% FBS/PBS for 1 h at RT. Following the same wash step as above, BCIP/NBT‐plus substrate was added to each well to allow visualisation of alkaline phosphatase activity. Plates were incubated until distinct spots emerged on the filter, termed spot forming units (SFU), at which point excess BCIP/NBT was removed by extensively washing the plate with dH2O. Spots were counted with the AID 5000 pro XI ELISpot reader.

### Clonogenic lung metastases assay

At the respective experimental endpoints, mice were euthanised and the lungs and tumors harvested, weighed, and placed into 1 mL of Hank's buffered saline solution (HBSS; Gibco, Grand Island, NY, USA). Using aseptic conditions, lungs were cut into small pieces, incubated with the digestion mix of liberase TL and DNase I (both Roche, Mannheim, Germany) for 45 min at 37 °C, and processed through a 70‐μm filter. After centrifugation at 572 *g* for 4 min, RBCs were lysed with RBC lysis solution and samples were washed twice in 10 mL of HBSS. Tumors were processed through a 70‐μm filter, centrifuged at 572 *g* for 4 min, and washed twice with HBSS. Following all wash steps, cells were resuspended in 10 mL cRPMI medium, supplemented with 10 μg mL^−1^ 6‐TG. Lung cells were diluted 10‐, 100‐, 1000‐, 10 000‐fold in 10 mL of cRPMI medium with 6‐TG and transferred alongside neat lung samples onto 10 cm tissue culture dishes (Corning). Approximately 200 μg of each tumor was plated to determine what percentage of 6‐TG resistant tumor cells had retained GFP and by extension HER2 expression. Lung plates were incubated undisturbed at 37 °C, 5% CO_2_ for 14 days. For tumors, a wash step was performed at day 1 of incubation, with the medium discarded and fresh cRPMI medium with 6‐TG added. After the 14‐day incubation, the culture medium was discarded, and colonies fixed for 5 min in methanol. Plates were then washed with dH2O, stained with 0.03% methylene blue for 5 min and washed once more in dH2O. Plates were left to dry before the stained colonies were counted and images taken. The number of tumor colonies per gram of lung tissue was then calculated.

### Histology and analysis

One lung lobe was collected per mouse and processed according to standard formalin‐fixed paraffin‐embedded protocol. Histological data were collected from four haematoxylin and eosin (H&E)‐stained sections from each mouse using a whole slide scanner VS200 (Olympus, Tokyo, Japan) equipped with APOPLAN 20X objective NA 0.75 and Hamamatsu ORCA‐Flash 4 V3 sCMOS camera. Sections were 5 μm thick and were first collected at the lung surface and then serially every 300 μm so that depth analysis was approximately 900 μm though the tumor. Images were recorded using Olympus VS200 ASW software (version 3.3) in standard brightfield settings. Images were analysed using QuPath software version 0.3.0. (Bankhead *et al*. 2017) by an observer blinded to the clonogenic assay results (AS). First, a simple tissue selection was applied to identify the whole lung section and remove artefacts from the analysis. Metastatic areas were then identified using the pixel classification tool trained using annotations extracted from metastatic regions from multiple image sections and with an area size classification of 100–500 μm^2^ included. Algorithm performance and selection confirmed by eye with manual adjustments made to the algorithm to remove false‐positive selection of crushed lung areas or venules. The data set was tailored using Excel (Microsoft version 16.60), and plots were generated using Prism 9.0 (Version 9.3.1).

### Statistical analysis and data availability

Figures were generated and statistical analysis performed using GraphPad Prism 8 software (Graphpad Prism Inc). Data are shown are mean values ± SEM as indicated in the figure captions. A Mann–Whitney *U*‐test was used for analysis of only two groups. For more than two unpaired groups, one‐way analysis of variance (ANOVA) was performed before Tukey's multiple comparison test. For paired data, a two‐way ANOVA was performed before Tukey's or Sidak's multiple comparison test. For survival curves, a Gehan‐Breslow‐Wilcoxon test was used. In all experiments, *P*‐values ≤ 0.05 were taken to be significant. The data generated during the current study are available from the corresponding author on reasonable request.

## AUTHOR CONTRIBUTIONS

**Olivia Kelsen Burn:** Conceptualization; data curation; formal analysis; methodology; writing – original draft; writing – review and editing. **Kathryn Farrand:** Data curation; methodology. **Tara Pritchard:** Data curation; methodology. **Sarah Draper:** Data curation; methodology. **Ching wen Tang:** Data curation; methodology. **Anna H Mooney:** Data curation; methodology. **Alfonso J Schmidt:** Methodology; data curation. **Sung Hyun Yang:** Data curation; resources. **Geoffrey Williams:** Resources. **Margaret Brimble:** Resources; supervision. **Matheswaran Kandasamy:** Data curation; resources. **Andrew J Marshall:** Data curation; resources. **Kate Clarke:** Conceptualization; funding acquisition. **Gavin Painter:** Conceptualization; funding acquisition. **Ian Hermans:** Conceptualization; formal analysis; supervision. **Robert Weinkove:** Conceptualization; formal analysis; funding acquisition; supervision; writing – original draft; writing – review and editing.

## Conflicts of interests

IH and GP are inventors on patents licensed to or owned by biotech start‐up Avalia Immunotherapies Limited.

## Ethical approval and consent to participate

Animal ethics approval was sort from Victoria University of Wellington Animal Ethics Committee (application approval number 26384). Consent to participate is not applicable for this manuscript.

### Data availability statement

All data relevant to the study are included in the article or uploaded as online supplementary information. The data presented in this report are available from the corresponding author on reasonable request.

## Supporting information


Supporting Information
