# Supplementary material for: Glycolipid‐peptide conjugate vaccines elicit CD8 + T‐cell responses and prevent breast cancer metastasis
Source: Clin Transl Immunology. 2022 Jul 3;11(7):e1401. doi: 10.1002/cti2.1401 (PMC9250805; doi:10.1002/cti2.1401)

Supplementary table 1. Antibody panel used for NKT cell flow cytometry analysis.

Marker	Fluorophore	Identifier (clone, RRID)	Source	Dilution
CD1d tetramer	BV421	PBS-57-loaded CD1d tetramer	NIH tetramer facility	0.5 µL/sample
TCR-β	FITC	Conjugated in-house		1:500
CD161 (NK1.1)	BV650	PK136, AB_11147949	BioLegend	1:500
CD64	AF647	X54-5/7.1, AB_2566560	BioLegend	1:300
CD19	AF647	1D3	BD Biosciences	1:300
CD44	BV750	IM7, AB_2871973	BD Biosciences	1:600
CD69	PerCP-Cy5.5	H1.2F3, AB_394051	BD Biosciences	1:200
CD279 (PD-1)	PE-Cy7	29F-1A12, AB_10696422	BioLegend	1:500
KLRG1	APC	2F1/KLRG1, AB_10645509	BioLegend	1:900
Viability	ZOMBIE™ NIR		BioLegend	1:1000

Supplementary table 2. Antibody panel used for APC flow cytometry analysis.

Marker	Fluorophore	Identifier (clone, RRID)	Source	Dilution
MHCII I-Ab	eF450	AF6-120.1; AB_10669941	eBioScience	1:1000
CD3ε	FITC	UCHT1, AB_314060	BioLegend	1:600
CD11b	BB700	M1/70, AB_2744272	BD BioScience	1:800
CD11c	BV785	N418, AB_2565268	BioLegend	1:100
CD86	APC-R700	GL1, AB_2739258	BD BioScience	1:800
CD64	AF647	X54-5/7.1, AB_2566560	BioLegend	1:300
B220	PerCP	RA3-6B2, AB_893355	BD BioScience	1:200
Viability	Zombie^{TM} NIR		BioLegend	1:1000

Supplementary table 3. Antibody panel used for T cell analysis.

Marker	Fluorophore	Identifier (clone, RRID)	Source	Dilution
OVA pentamer	PE	$OVA_{257-264}$ peptide-loaded H-2K^b pentamer	ProImmune	0.5 µL/sample
HER2 tetramer	PE	H-2K^d/HER2$_{63-71}$ tetramer complex	NIH tetramer facility	1 µL/sample
CD3ε	BV421	145-2C11, AB_10898314	BioLegend	1:200
CD8α	AF700	53-6.7, AB_494005	eBioScience	1:600
B220	PerCP	RA3-6B2, AB_893355	BD BioScience	1:200
CD64	AF647	X54-5/7.1, AB_2566560	BioLegend	1:300
CD44	BV711	IM7, AB_2564214	BioLegend	1:800
Viability	Zombie™ NIR		BioLegend	1:1000

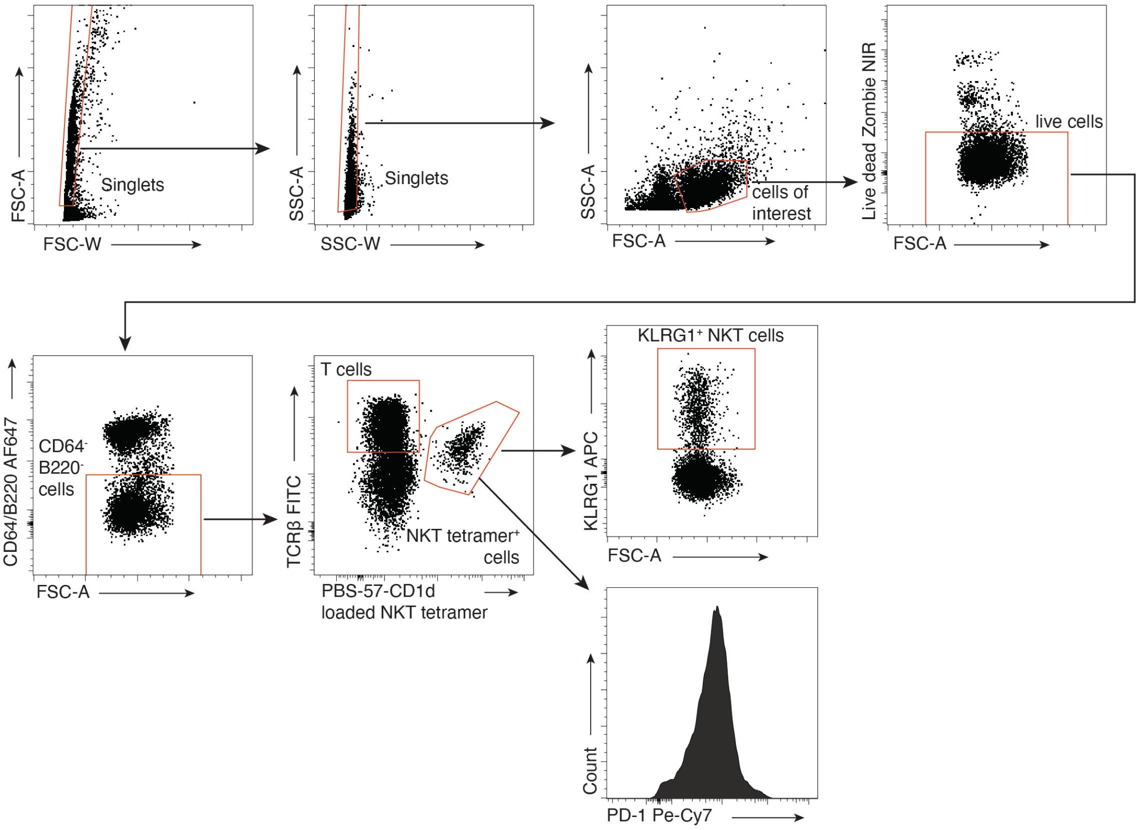

Supplementary figure 1. Gating strategy for NKT cells. Treatment and tissue collection was as described for figure 1. Antibodies used are listed in Supplementary table 1. The gating example shown is on splenocytes from an α-GalCer-OVA treated animal. After gating on singlets and cells of interest (the gate was set in the lymphocytes region using the scatter profile of the cells), live cells were defined by staining with live dead Zombie NIR. Then the strategy shown was used to identify T cells (TCR-β^{+} PBS-57-loaded CD1d tetramer^{-} cells) and NKT cells (TCR-β^{+} PBS-57-loaded CD1d tetramer^{+} cells). The MFI of PD-1 was then identified on these two subsets and the frequency of NKT cells positive for KLRG1 determined.

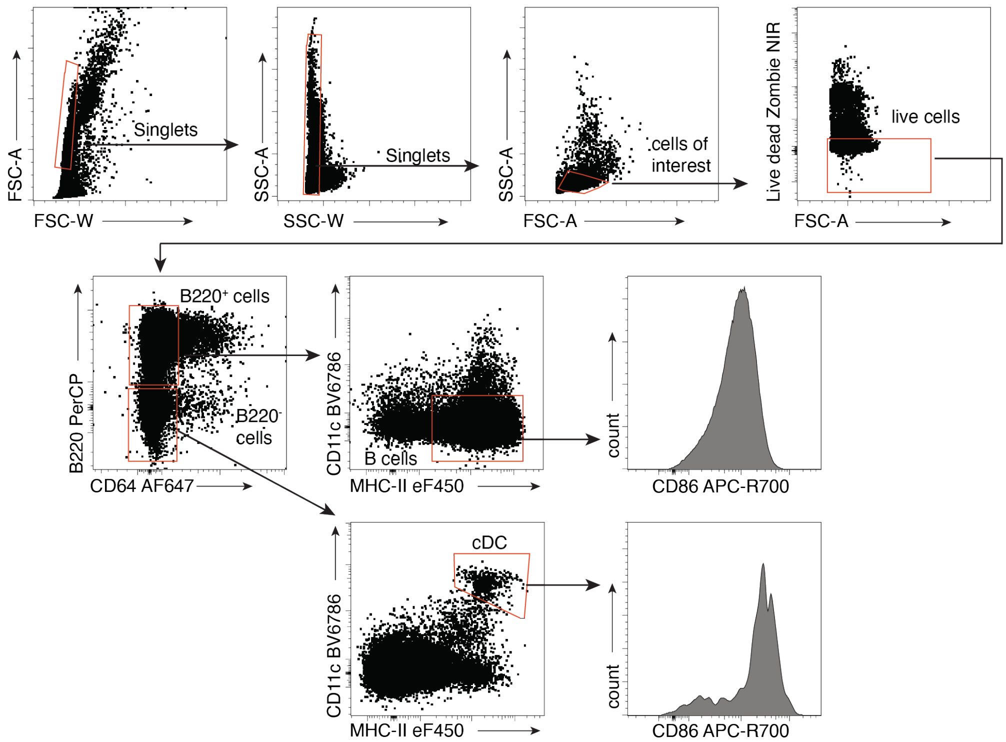

Supplementary figure 2. Gating strategy for APCs. Treatment and tissue collection was as described for Figure 2. Antibodies used are listed in supplementary table 2. The gating example shown is on splenocytes from control and α-GalCer-HER2 treated animals. After gating on singlets and cells of interest (the gate was set in the lymphocytes region using the scatter profile of the cells), live cells were defined by staining with live dead zombie NIR. Then the strategy shown was used to identify B cells ($B220^+$ cells), cDCs ($B220^-$, $CD64^-$, $CD11c^+$, $MHC\text{-}II^+$). The MFI of CD86 was then identified on these subsets, as shown on the histograms.

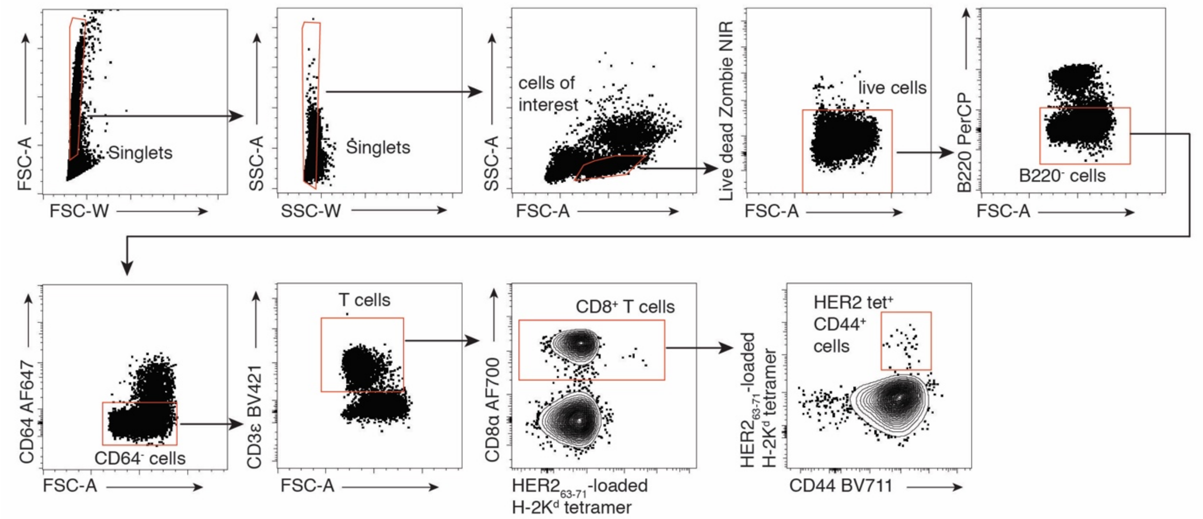

Supplementary figure 3. Gating strategy for HER2^{+} CD44^{+} CD8^{+} T cells. Treatment and tissue collection was as described for Figure 2. Antibodies used are listed in Supplementary table 3. The gating example shows day 7 peripheral blood from an α-GalCer-HER2 treated animal. Antigen-specific CD8^{+} T cells were defined as HER2$_{63\text{-}71}$-loaded H-2K^{d} tetramer^{+} CD44^{+} CD8^{+} CD3ε^{+} CD64^{-} B220^{-} live lymphocytes.

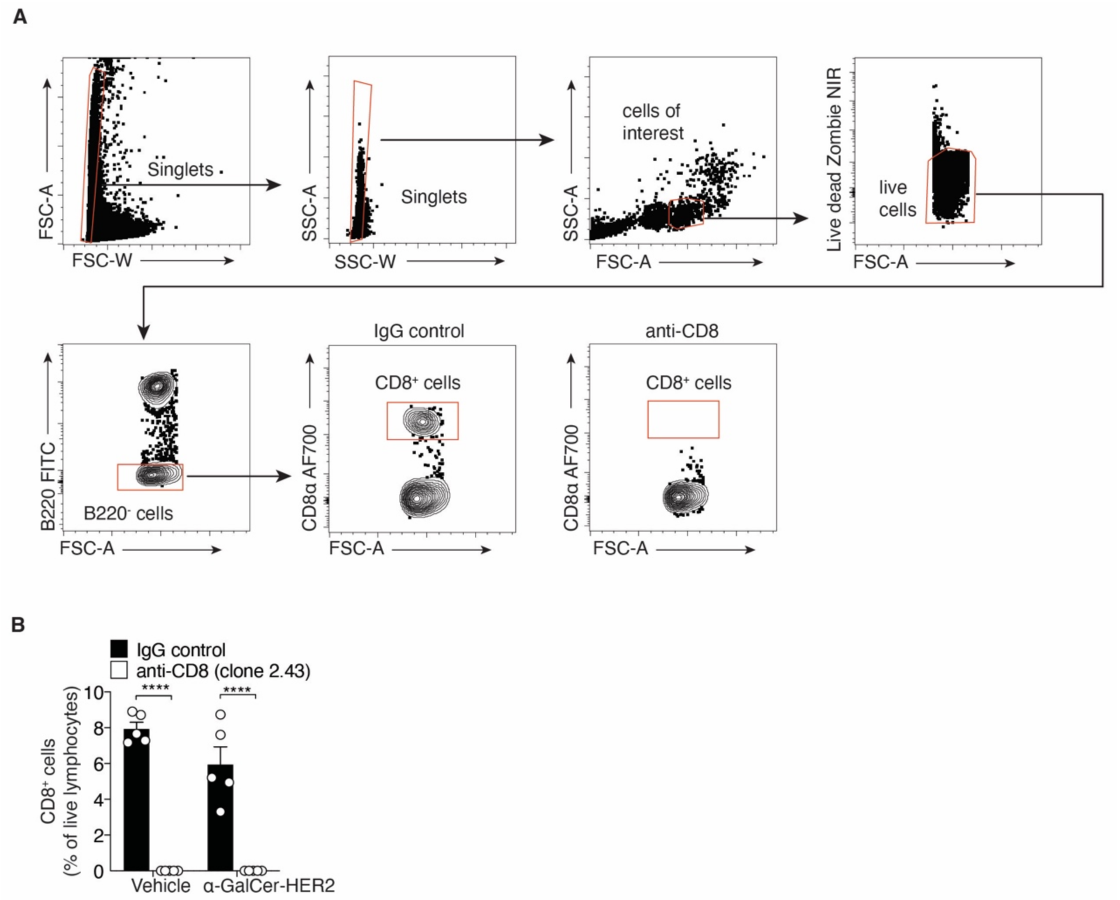

Supplementary figure 4. Depletion of CD8^{+} cells using monoclonal anti-CD8 antibody. Treatment and tissue collection was as described for Figure 3. (A) The gating example shown in on peripheral blood from α-GalCer-HER2 treated animals administered either IgG control or the anti-CD8 monoclonal antibody (clone 2.43) intraperitoneally day 5 and 6 after vaccination. Samples were collected on day 7 post-vaccination. After gating on singlets and cells of interest (the gate was set in the lymphocytes region using the scatter profile of the cells), live cells were defined by staining with live dead Zombie NIR. Then the strategy shown was used to identify CD8^{+} cells (B220^{-}CD8^{+}). (B) The frequency of CD8^{+} cells in the peripheral blood across the two treatment groups in mice treated with either control or α-GalCer-HER2.

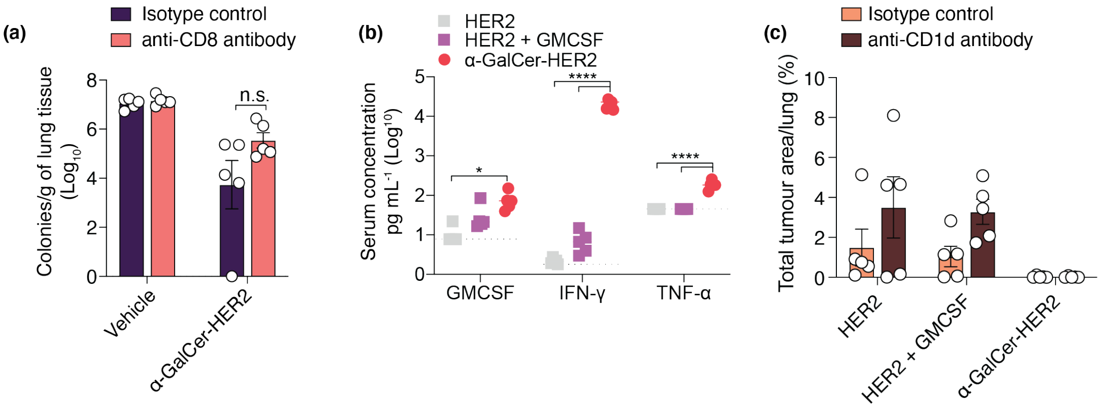

Supplementary figure 5. Response to blockade of CD8$^+$ T cell and NKT cell activity. (a) Mice shown in **Figure 4d** were challenged I.V. with 4T1.2-HER2 tumours cells 7 days after challenge with the syngeneic splenocytes (day 14 post vaccination) and tumour colonisation of the lungs assessed 12 days later. Shown is mean number of colonies per gram of lung tissue for each treatment group (n = 5). **(b)** Serum cytokines at 18 hr after the administration of α-GalCer-HER2 vaccine or HER2 peptide alone or in combination with GM-CSF. The dotted line indicates the limit of detection for each cytokine, respectively. **(c)** Percentage of the lung area positive for tumour metastasis. Shown is the sum of the area from all four sections taken per lung with n=5 mice per treatment group. n.s. *P>0.05;* **P* < 0.05; *****P* < 0.0001; **(a–c)** one-way ANOVA with Tukey's multiple comparison test.

Supplementary methods

Peptide Synthesis

Aminooxyacetyl-FFRK-TYLPTNASL

Aminooxylacetyl-FFRK-TYLPTNASL was synthesised by Fmoc SPPS on a 4-(4-hydroxymethyl-3-methoxyphenoxy)butyric acid (HMPB) ChemMatrix® resin preloaded with Fmoc-L-Leucine (Fmoc-Leu) as the first amino acid. α-Amino acids with the following side-chain protecting groups were used: Arg(Pbf), Lys(Boc), Ser(tBu), Thr(tBu), Asn(Trt), Tyr(tBu), and the peptide was synthesised using a Biotage® Initiator+ Alstra™ microwave peptide synthesiser on a 0.1 mmol scale. The resin was swelled in DMF (20 min, 70 °C, 50 W) followed by synthesis reaction cycles consisting of: Fmoc deprotection with 20% piperidine in DMF (3 min and then 10 min, rt); and amino acid coupling (5 min, 75 °C, 50 W) employing 5 equivalents of the protected amino acid in DMF (0.5 M) activated by 5 equivalents of diisopropylcarbodiimide and Oxyma® (both 0.5 M in DMF). Fmoc-Arg(Pbf) and was coupled for 60 min at rt. N-Boc-aminooxyacetic acid N-hydroxysuccinimide ester (3 equivalents in 3 mL DMF) was incorporated at the N-terminus using collidine (3 equivalents) following the final Fmoc deprotection. The resin was washed with CH_2Cl_2 and dried under vacuum. The subsequent cleavage from the resin was achieved by incubating the resin in 10 mL of 88:5:5:2:1 TFA/water/phenol/i-Pr_3SiH/aoAA-HCl at 0 °C for 10 min. The resin was filtered, rinsed with a further 10 mL of the cleavage solution and left to stand at room temperature for 2 h. Crude peptide was precipitated and triturated with cold diethyl ether, isolated (centrifugation), and lyophilized from 95:5:0.2 water/MeCN/TFA.

Semi-preparative HPLC purification was conducted employing an Agilent 1260 Infinity HPLC system fitted with a Phenomenex Gemini C18 5µm 110 Å 10 x 250mm column, with water/0.1% TFA as eluent A and MeCN/0.1% TFA as eluent B. Approximately 170 mg of crude peptide was dissolved to a concentration of 2.5 mg mL^{-1} in 5% MeCN in water and loaded in its entirety onto the column. A shallow gradient from 17% B to 27% B over 100 min was generated and fractions collected at 1 minute intervals. The fractions were analysed by HPLC, with those containing the desired peptide at sufficient purity being pooled and lyophilized. The purified peptide showed a main peak for the target peptide (93%) with a retention time of 6.8 min, and minor impurities. The mass signal at m/z 1631.4 (calculated 1631.8 for $[M+H]^{1+}$) confirmed the identity of the major product.

Aminooxyacetyl-FFRK-KISQAVHAAHAEINEAGRESIINFEKLTEWT

Aminooxylacetyl-FFRK-KISQAVHAAHAEINEAGRESIINFEKLTEWT was synthesised on a 0.1 mmol scale using the Tribute® automated peptide synthesiser (Gyros Protein Technologies) employing standard Fmoc-Solid-supported peptide synthesis techniques. TentaGel S® aminomethyl resin was derivatized with Fmoc-L-threonyl-4-(oxymethyl)phenoxypropionic acid (Fmoc-L-Thr-MPPA) and the Fmoc group removed (20% piperidine in DMF). Subsequent couplings were performed with Fmoc-protected α-amino acids; those residues requiring side-chain protecting groups were as follows: Arg(Pbf), Asn(Trt), Gln(Trt), Glu(tBu), His(Trt), Lys(Boc), Ser(tBu), Tyr(tBu) and Trp(Boc). The introduction of amino acid pairs, Glu-Ser (ES) and Leu-Thr (LT) was achieved using Fmoc-pseudoproline dipeptides Fmoc-Glu(tBu)-Ser($\psi^{Me,Me}$pro)-OH and Fmoc-Leu-Thr($\psi^{Me,Me}$pro)-OH, respectively. Fmoc-Arg(Pbf), Fmoc-Gln(Trt) and Fmoc-Val were double-coupled. On

completing the addition of each Fmoc amino acid residue to the peptide, the resin was treated with acetic anhydride (20% in DMF, 1 minute). Fmoc protecting groups were then removed with 20% piperidine in DMF (10 min twice). The peptide was elaborated by an iterative series of peptide couplings as follows: a solution of the Fmoc-protected amino acid (5 equivalents relative to resin) and O-(7-azabenzotriazol-1-yl)-N,N,N′,N′-tetramethyluroniumhexafluoro phosphate (HATU, 0.49 equivalents) in DMF (approx. 2.0 mL) was activated by the addition of 4-methylmorpholine (10 equivalents) and transferred to the resin, which was agitated at rt for 60 minutes. The final coupling step entailed incubating the resin for 60 minutes with a solution comprised of N-Boc-aminooxyacetic acid N-hydroxysuccinimide ester and collidine (3 equivalents of each in 2 mL DMF). The resin was washed with CH_2Cl_2 and air-dried. Cleavage of the peptide from resin was achieved using 5 mL of 91.5:5:2.5:1 TFA/water/ethanedithiol/iPr_3SiH at rt for 2 h. The resin was filtered and washed with a further 3 mL of TFA. The combined filtrates were diluted with chilled diethyl ether (30 mL) and the resulting precipitated, crude peptide pelleted by centrifugation. The pellet was washed (ether), air-dried, dissolved in 1:1 MeCN/water containing 0.1% TFA (15 mL) and heated at 65˚C for 30 minutes before being lyophilized.

Purification was carried out using a Thermo Scientific Dionex Ultimate 3000 HPLC system fitted with a Phenomenex Gemini C18 (5μm 110 Å) 10×250 mm column and using with water/0.1% TFA as eluent A and MeCN/0.1% TFA as eluent B. Batch purification of 10 mg of peptide dissolved to a concentration of 2.0 mg mL^{-1} in 10% MeCN in water (0.1% TFA) and loaded in on to the column and eluted using a gradient from 1-25% B over 3 min followed by 25-55% B over 40 min, collecting those fractions with desired material.

The material obtained from repeated runs was pooled and lyophilised. Analytical HPLC using a Phenomenex Gemini C18 column (5μm 110 Å) 4.6×150 mm column, the same eluents as above and a gradient of 5%B to 65%B over 60 minutes established a purity of >98% with a retention time of 33.8 min.

The identity of the product was confirmed by mass spectrometry: found (m/z) 1382.6 $[M+3H]^{3+}$; calculated 1382.87 $[M+3H]^{3+}$.

Aminooxyacetyl-FFRK-GARGPESRLLEFYLAMPFATPMEAELARRSLAQDAPPL

Aminooxyacetyl-FFRK-GARGPESRLLEFYLAMPFATPMEAELARRSLAQDAPPL was synthesised on a 0.1 mmol scale using the Tribute® automated peptide synthesiser (Gyros Protein Technologies) employing standard Fmoc-Solid-supported peptide synthesis techniques. TentaGel S® aminomethyl resin was derivatized with Fmoc-L-Leucinyl-4-(oxymethyl)phenoxypropionic acid (Fmoc-L-Leu-MPPA) and the Fmoc group removed (20% piperidine in DMF). Subsequent couplings were performed with Fmoc-protected α-amino acids; those residues requiring side-chain protecting groups were as follows: Arg(Pbf), Asp(tBu), Gln(Trt), Glu(tBu), Lys(Boc), Ser(tBu), Thr(tBu) and Tyr(tBu). The Ala-Thr amino acid pair was introduced using the corresponding Fmoc-pseudoproline dipeptide Fmoc-Ala-Thr($\psi^{Me,Me}$pro)-OH. The first six residues of the synthesis (the C-terminal portion of the peptide) were added using single couplings; all other residues were double-coupled. On completing the addition of each Fmoc amino acid residue to the peptide, the resin was treated with acetic anhydride (20% in DMF, 1 minute). Fmoc protecting groups were then removed with 20% piperidine in DMF (10 min twice). The peptide was elaborated by an iterative series

of peptide couplings as follows: a solution of the Fmoc-protected amino acid (5 equivalents relative to resin) and O-(7-Azabenzotriazol-1-yl)-N,N,N′,N′-tetramethyluroniumhexafluoro phosphate (HATU, 0.49 equivalents) in DMF (2.0 mL) was activated by the addition of 4-methylmorpholine (10 equivalents) and transferred to the resin, which was agitated at rt for 60 minutes (this process was repeated for double couplings). The final coupling step entailed incubating the resin for 60 minutes with a solution comprised of N-Boc-aminooxyacetic acid N-hydroxysuccinimide ester and collidine (3 equivalents of each). The resin was washed with CH_2Cl_2 and air-dried. Cleavage of the peptide from resin was achieved using 5 mL of 91.5:5:2.5:1 TFA/water/ethanedithiol/iPr_3SiH at rt for 2 h. The resin was filtered and washed with a further 3 mL of TFA. The combined filtrates were diluted with chilled diethyl ether (30 mL) and the resulting precipitated, crude peptide pelleted by centrifugation. The pellet was washed (ether), air-dried, dissolved in 1:1 MeCN/water containing 0.1% TFA (15 mL) and heated at 65˚C for 30 minutes before being lyophilized.

Purification was carried out using a Thermo Scientific Dionex Ultimate 3000 HPLC system fitted with a Phenomenex Gemini C18 (5 µm 110 Å) 10×250 mm column and using with water/0.1% TFA as eluent A and MeCN/0.1% TFA as eluent B. Crude peptide was dissolved in water/MeCN to 10 mg mL^{-1} and 500 µL aliquots purified using linear gradients of 5-30%B over 3 min followed by 30-42%B over 16 minutes, collecting the desired component. The material obtained from repeated runs was pooled and lyophilised. Analytical HPLC using a waters Xterra C18 4.6×150mm column, the same eluents as above and a gradient of 5%B to 65%B over 20 minutes established a purity of >97% with a retention time of 15.3 min.

The identity of the product was confirmed by mass spectrometry: found (m/z) 1609.1 $[M+3H]^{3+}$; calculated 1609.2 $[M+3H]^{3+}$

Conjugate vaccine synthesis

Oxime conjugate vaccine α-GalCer-OVA

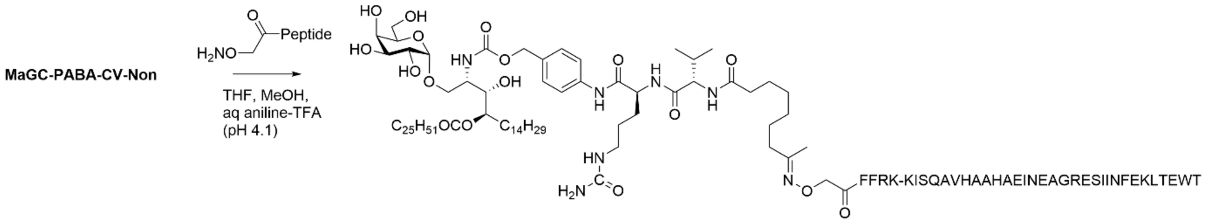

Aniline buffer (pH = 4.1, 300 mM) was prepared by mixing freshly distilled aniline (5.5 mL) and TFA (3.9 mL) in MilliQ water, and making up to a total volume of 200 mL. THF was distilled from 2,4-dinitrophenylhydrazine. A mixture of peptide aminooxyacetyl-FFRK-KISQAVHAAHAEINEAGRESIINFEKLTEWT (8.8 mg, 2.1 µmol) and ketone MaGC-PAB-CV-Non (1.9 mg, 1.3 µmol) was heated in 4:2:3 THF/MeOH/aniline buffer (300 µL) at 50 °C for 16 h. The product mixture was purified by preparative HPLC [Phenomenex Luna C18(2), 5 µm, 250 x 21.2 mm, 30 °C, 17 mL/min; Mobile phase A = 30:70:0.05 water/MeOH/TFA; Mobile phase B = 100:0.05 MeOH/TFA; 0-13 min: 100% A to 100% B; 13-15 min: 100% B; 15-16 min: 100% B to 100% A; 16-18 min: 100% A] to give the title compound as a white

solid (4.7 mg, 99%). HRMS-ESI m/z calculated for $C_{264}H_{425}N_{59}O_{70}$ $[M+4H]^{4+}$ 1387.2877, found 1387.3015.

HPLC-MS-CAD

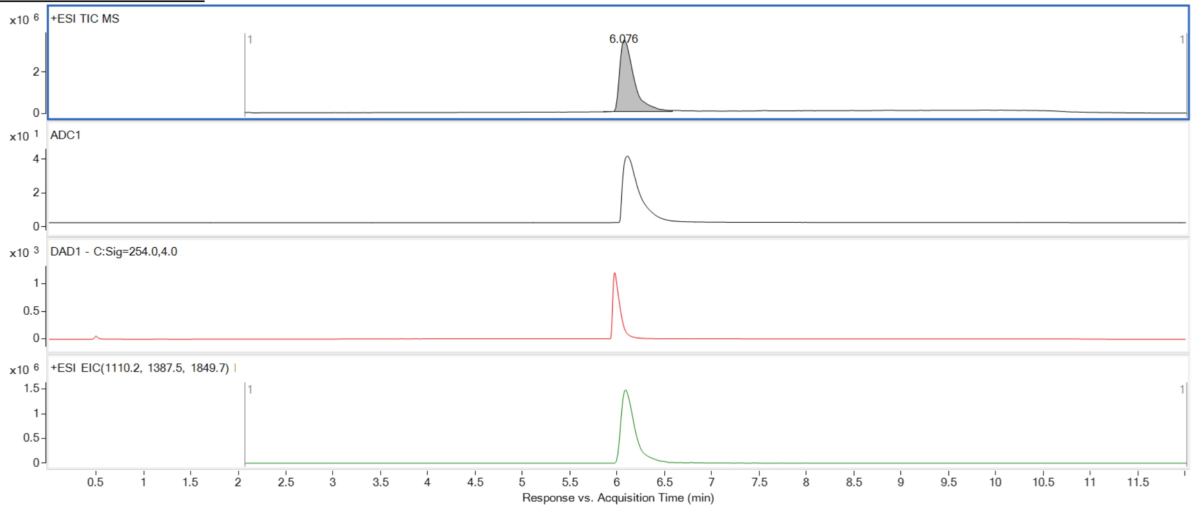

Extracted MS from shaded peak in TIC:

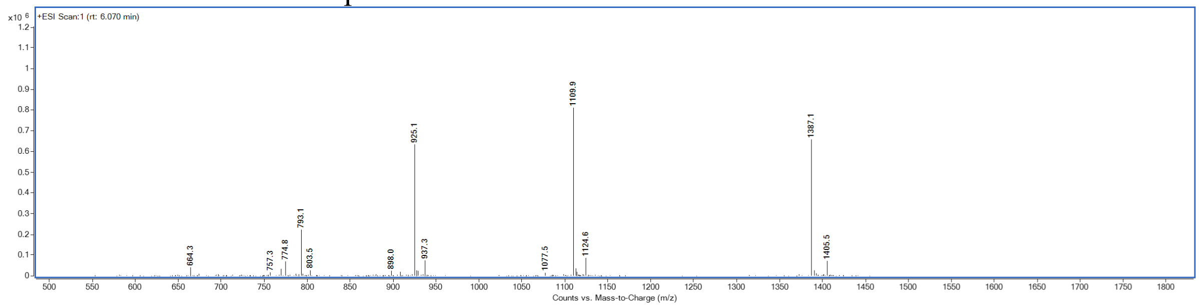

Oxime conjugate vaccine α-GalCer-NY-ESO-1

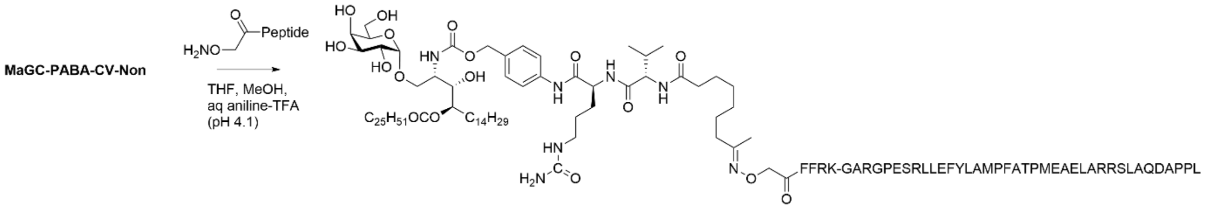

Aniline buffer (pH = 4.1, 300 mM) was prepared by mixing freshly distilled aniline (5.5 mL) and TFA (3.9 mL) in MilliQ water, and making up to a total volume of 200 mL. THF was distilled from 2,4-dinitrophenylhydrazine. A mixture of peptide aminooxyacetyl-FFRK-GARGPESRLLEFYLAMPFATPMEAELARRSLAQDAPPL (7.32 mg, 1.5 µmol) and ketone MaGC-PAB-CV-Non (1.4 mg, 1.0 µmol) was heated in 4:2:3 THF/MeOH/aniline buffer (300 µL) at 50 °C for 16 h. The product mixture was purified by preparative HPLC [Phenomenex Luna C18(2), 5 µm, 250 x 21.2 mm, 30 °C, 17 mL/min; Mobile phase A = 30:70:0.05 water/MeOH/TFA; Mobile phase B = 100:0.05 MeOH/TFA; 0-13 min: 100% A to 100% B; 13-15 min: 100% B; 15-16 min: 100% B to 100% A; 16-18 min: 100% A] to give the title compound as a white solid (2.9 mg, 99%). HRMS-ESI m/z calculated for $C_{296}H_{478}N_{66}O_{75}S_2$ $[M+6H]^{6+}$ 1037.9255, found 1037.9252.

HPLC-MS-CAD

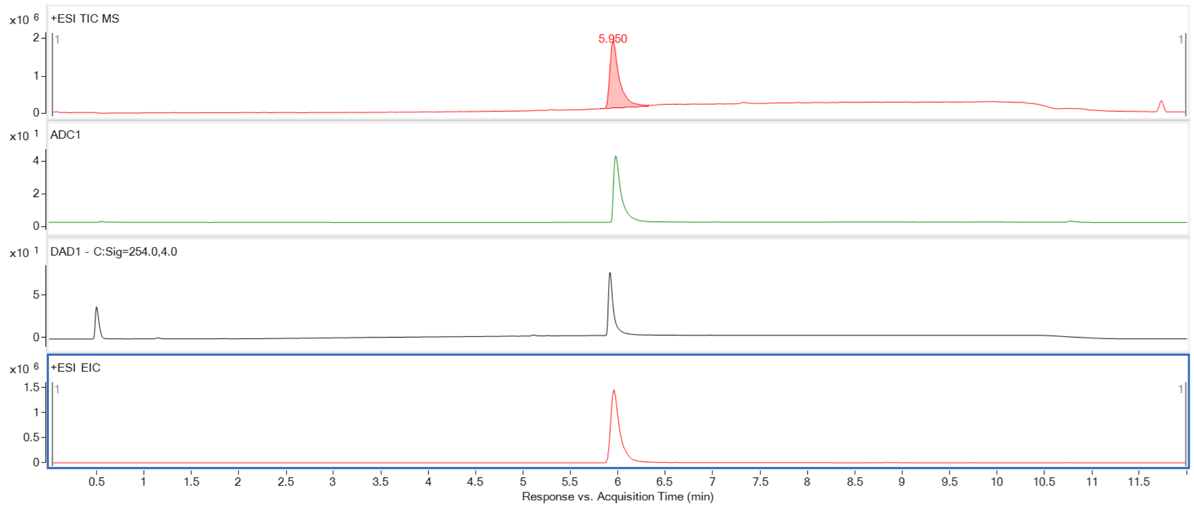

Extracted ion from shaded peak in TIC:

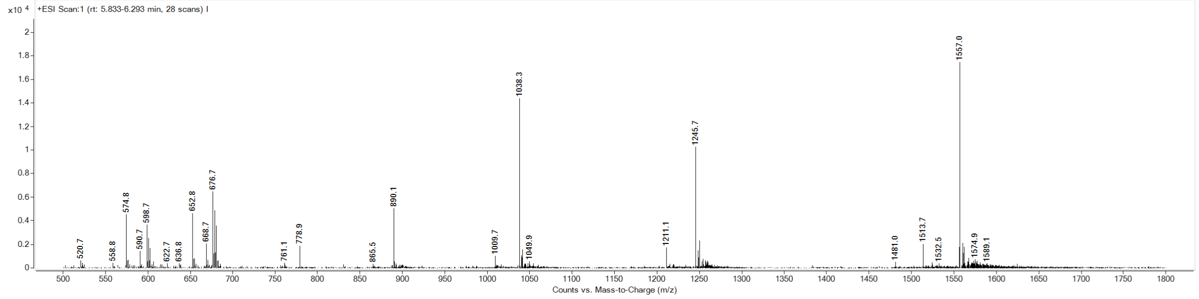

Oxime conjugate vaccine α-GalCer-HER2

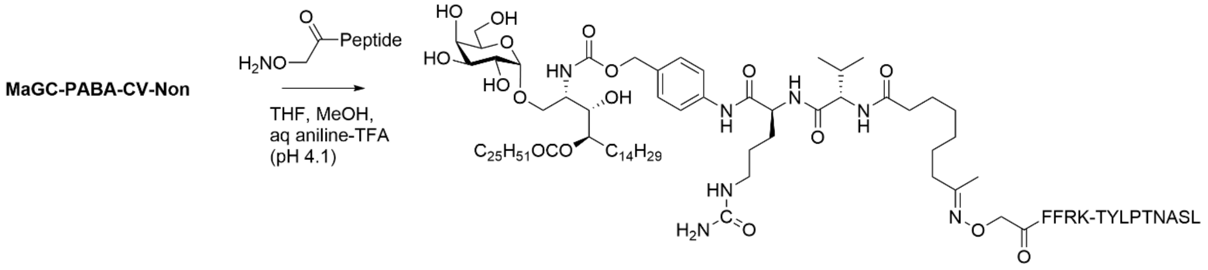

Aniline buffer (pH = 4.1, 300 mM) was prepared by mixing freshly distilled aniline (5.5 mL) and TFA (3.9 mL) in MilliQ water, and making up to a total volume of 200 mL. THF was distilled from 2,4-dinitrophenylhydrazine. A mixture of peptide aminooxyacetyl-FFRK-TYLPTNASL (3.9 mg, 2.4 µmol) and ketone MaGC-PAB-CV-Non (2.0 mg, 1.4 µmol) was heated in 4:2:3 THF/MeOH/aniline buffer (300 µL) at 50 °C for 16 h. The product mixture was purified by preparative HPLC [Phenomenex Luna C18(2), 5 µm, 250 x 21.2 mm, 30 °C, 17 mL/min; Mobile phase A = 30:70:0.05 water/MeOH/TFA; Mobile phase B = 100:0.05 MeOH/TFA; 0-13 min: 100% A to 100% B; 13-15 min: 100% B; 15-16 min: 100% B to 100% A; 16-18 min: 100% A] to give the title compound as a white solid (2.9 mg, 99%). HRMS-ESI m/z calculated for $C_{154}H_{253}N_{25}O_{36}$ $[M+2H]^{2+}$ 1515.4446, found 1515.4438.

<u>HPLC-MS-CAD</u>

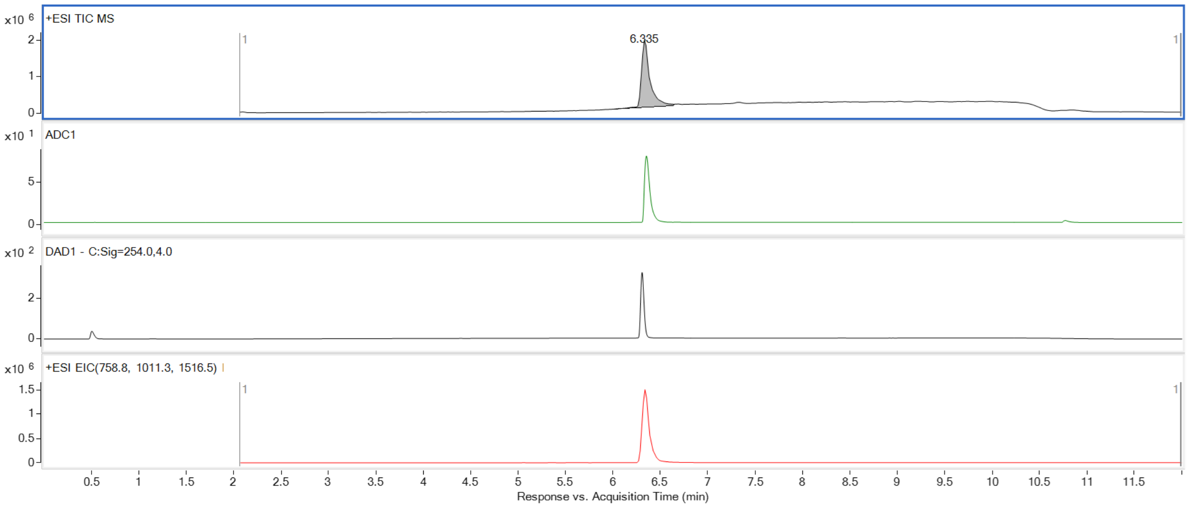

Extracted ion from shaded peak in TIC:

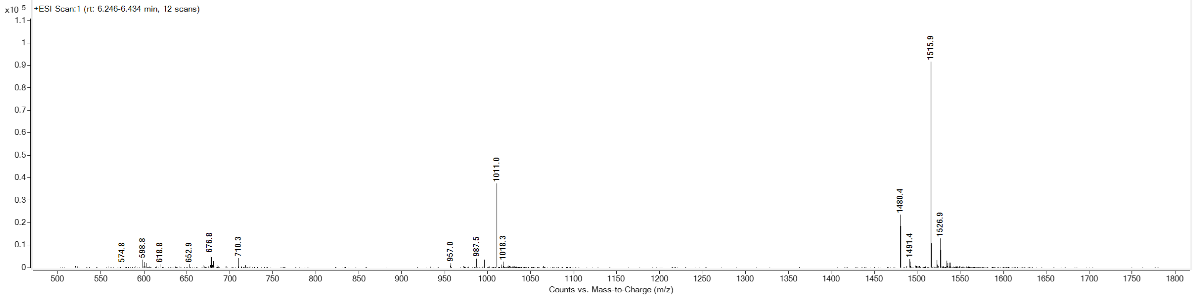

Supplement: Supplementary file 1 — Supporting Information [file CTI2-11-e1401-s001.pdf]
